# Drivers and Patterns of Ground-Dwelling Beetle Biodiversity across Northern Canada

**DOI:** 10.1371/journal.pone.0122163

**Published:** 2015-04-22

**Authors:** Crystal M. Ernst, Christopher M. Buddle

**Affiliations:** Department of Natural Resource Sciences, McGill University, Sainte-Anne-de-Bellevue, Quebec, Canada; Universidad de Granada, SPAIN

## Abstract

Many macroecological patterns of biodiversity, including the relationship between latitude and species richness, are well-described. Data collected in a repeatable, standardized manner can advance the discipline beyond the description of patterns and be used to elucidate underlying mechanisms. Using standardized field methods and a hyper-diverse focal taxon, viz. Coleoptera, we aim to (1) describe large-scale latitudinal patterns of taxonomic diversity, functional diversity, and assemblage structure across northern Canada, and (2) determine which climatic, spatial, and habitat variables best explain these patterns. We collected terrestrial beetles at twelve locations in the three northernmost ecoclimatic zones in North America: north boreal, subarctic, and high arctic (51–81°N, 60–138°W). After identifying beetles and assigning them to a functional group, we assessed latitudinal trends for multiple diversity indices using linear regression and visualized spatial patterns of assemblage structure with multivariate ordinations. We used path analysis to test causal hypotheses for species and functional group richness, and we used a permutational approach to assess relationships between assemblage structure and 20 possible climatic and environmental mechanisms. More than 9,000 beetles were collected, representing 464 species and 18 functional groups. Species and functional diversity have significant negative relationships with latitude, which are likely explained by the mediating effects of temperature, precipitation, and plant height. Assemblages within the same ecoclimatic zone are similar, and there is a significant relationship between assemblage structure and latitude. Species and functional assemblage structure are significantly correlated with many of the same climatic factors, particularly temperature maxima and minima. At a large spatial extent, the diversity and assemblage structure of northern beetles show strong latitudinal gradients due to the mediating effects of climate, particularly temperature. Northern arthropod assemblages present significant opportunities for biodiversity research and conservation efforts, and their sensitivity to climate make them ideal targets for long-term terrestrial diversity monitoring.

## Introduction

Macroecologists have successfully described large-scale spatial patterns of biodiversity and species distributions. Among them, the latitudinal gradient of species richness, in which fewer species are found at high latitudes compared to at the equator, has captivated researchers for many decades [1]. Over the past decade, there has been increased interest in elucidating the causal mechanisms behind latitudinal diversity patterns [2–5]. The search for likely mechanisms has been challenging, however, and although a broad range of climatic, evolutionary, biotic, and spatial hypotheses have been put forth, reviewed by [3], no single factor has been identified as a key mechanism. It is highly likely that the number of species found at different latitudes, and the way these species assemble over space and time, is the result of multiple interacting ecological and evolutionary factors [6, 7]. Despite the challenge of teasing apart the relative contributions of different factors to patterns of diversity, climate—in particular, temperature—has been broadly recognized as a key element in both terrestrial and aquatic systems [8–10] and is worthy of additional testing.

Recently, some workers have begun including alternative or complimentary genetic, morphological or functional measures of diversity alongside the traditional taxonomic metric of species richness [11]. Function may play a particularly important role in influencing species diversity patterns, as species richness may be limited by biotic interactions, differences in life history traits, and environmental gradients that influence niche availability [12]. The inclusion of functional diversity in macroecological studies may ultimately yield stronger tests of biodiversity theories [5, 12, 13].

In addition to expanding the lens through which we examine diversity patterns beyond taxonomy, we can also use the framework established by earlier macroecological studies as a platform from which to broaden the scope of future work. Some authors have identified avenues of research that hold great promise for the advancement of macroecological theory, namely, the inclusion of: (1) standardized, repeatable faunal sampling, (2) broader taxonomic or functional scopes, (3) broader ecological scopes (e.g., multiple habitats or biomes), and/or (4) underrepresented yet ecologically significant biomes (aquatic systems and polar regions) and taxa (invertebrates, non-vascular plants and fungi) [3, 5]. While some of these advancements present logistical challenges, working to overcome them may generate sufficient quantitative data to test and support generalizable statements about large-scale patterns and processes of diversity [14, 15]. Understanding the underlying processes responsible for patterns of diversity will provide powerful mechanisms for predicting the effects of climate change and other anthropogenic disturbances on biotic communities and their component species [5].

Testing large-scale biodiversity patterns and processes requires using a focal taxon that is diverse, easily sampled, representative of different processes and functions, taxonomically well understood, and sensitive to environmental or ecological changes [16]. Beetles are an ideal study taxon: they are one of the most taxonomically and functionally diverse groups of animals [17], and are the most abundant, diverse and ecologically significant epigeic (i.e., living predominantly on the ground surface) insect taxon in northern systems [18, 19]. Beetles are also easily caught using passive trapping methods that can be standardized and thus permit comparisons of assemblages across time and space [20–22], and show rapid responses to environmental change [23–25]. These factors make northern beetles ideal focal taxa for conducting a comprehensive examination of terrestrial diversity and assemblage structure.

The overall objective of our research was to conduct a standardized, species-level assessment of both taxonomic and functional biodiversity patterns across multiple biomes, using beetles as a focal taxon. Our specific goals were to: (1) quantify latitudinal patterns in diversity and assemblage structure (taxonomic and functional), and (2) establish the climatic and/or environmental factors associated with taxonomic and/or functional assemblage structure across a large geographic extent, which was twelve locations in the three northernmost ecoclimatic zones of North America (Table 1). We hypothesized that: (1) beetles will conform to classical latitudinal gradients of diversity, with greater species and functional richness at lower latitudes; (2) species richness is directly influenced by climatic variables, which are influenced by latitude; (3) species and functional assemblage structures will display latitudinal gradients of similarity; (4) variations in assemblage structure will be best explained by climatic, rather than spatial or biotic, variables.

**Table 1:** Names, coordinates, and ecoclimatic zones of sampling locations, and dates of sampling.

**Sampling Location**	**Latitude, Longitude**	**Ecoclimatic Zone**	**Sampling Periods (dates)**			**Sampling Year**
			1	2	3	
Lake Hazen, NU	81.82975, -71.32244	High Arctic	19–23.vii	23–28.vii	-	2010
Banks Island, NWT	73.22412, -119.55255	High Arctic	7–11.vii	11–15.vii	15–19.vii	2011
Cambridge Bay, NU	69.12177, -105.41688	High Arctic	7–11.vii	11–15.vii	15–19.vii	2011
Iqaluit, NU	63.76144, -68.57352	High Arctic	17–21.vii	21–25.vii	25–29.vii	2010
Kugluktuk, NU	67.78157, -115.27824	Subarctic	22–26.vi	26–29.vi	29.vi-2.vii	2011
Tombstone Mtns., YT	64.60629, -138.35637	North Boreal	21–24.vi	24–27.vi	27.vi-01.vii	2011
Churchill, MB	58.73573, -93.79789	Subarctic	1–5.vii	5–9.vii	9–13.vii	2010
Schefferville, QC	54.75970, -66.71120	Subarctic	30.vi-3.vii	3–6.vii	6–9.vii	2010
Norman Wells, NWT	65.29112, -126.62262	Subarctic	7–11.vi	11–14.vi	14–17.vi	2011
Yellowknife, NWT	62.52110, -113.38174	North Boreal	7–11.vi	11–15.vi	15–18.vi	2011
Goose Bay, NFLD	53.21199, -60.45062	North Boreal	15–18.vi	18–21.vi	21–24.vi	2010
Moosonee, ON	51.28034, -80.64252	North Boreal	15–19.vi	19–23.vi	23–25.vi	2010

## Materials and Methods

### Study region and sampling locations

In 2010 and 2011, as part of a larger research project [26, 27] we collected terrestrial beetles at twelve locations in the three northernmost ecoclimatic regions of northern Canada [28]: four locations were in the north boreal region, four in the subarctic and four in the high arctic (Table 1). The extent of the study consists of a latitudinal gradient of 51–81°N and a longitudinal gradient of 60–138°W. Permits were granted by the following agencies to conduct sampling in the northern territories: Nunavut Research Institute (Scientific Research Licence), Nunavut Department of the Environment (Wildife Research Permit), Yukon’s Department of Tourism and Culture (Scientists and Explorers Licence), Aurora Research Institute of the Northwest Territories (Scientific Research Licence), Parks Canada Agency (Research and Collection Permit; for the Lake Hazen location, which falls in the Quttinirpaaq National Park of Canada, NU). In no instance did our work involve the collection endangered or protected species. In light of this fact, and since no other sampling was conducted in provincial parks or wildlife reserves, no specific permits were required to sample on public land in the other locations.

At each location, we established three replicates within about 15 km of each other. In consideration of the fact that habitat selection by northern arthropods is highly dependent on moisture and vegetation [29, 30], each replicate contained two broadly delimited and ecologically distinct habitats. “Mesic” habitats are characterized by higher elevations and well-drained soil, while “wet” habitats have saturated or poorly drained soils, and can be found in adjacent low-lying regions. The mesic vegetation was a discontinuous cover of dwarf shrubs, perennial forbs, and lichens. Wet habitats contained continuous cover of moss, sphagnum, saxifrages and sedges. In order to ensure consistency of sampling in both habitats across all locations, we established all replicates in open areas with no tree canopy cover; we encountered some dwarf black spruce in some of the more southern sites.

### Insect sampling and specimen processing

In all replicated habitats at each location, we set nine pitfall and nine yellow pan traps in three transects as a 30 x 75 m grid. We deployed 108 traps per location, and 1296 traps in the entire sampling design and we serviced them three times over two weeks (see Table 1 for collection dates, and for a complete description of trapping and collection protocols, see [26]). To account for phenology, we sampled the southernmost locations first, and the northernmost last, so that insect activity would be approximately equal at all locations. We placed specimens in 95% ethanol and returned them to the laboratory for sorting, and identified adult beetles to species or morphospecies using traditional morphological approaches.

We assigned each beetle to a functional group based on its predominant food source and mode of feeding [14]. Since biomass integrates functional characteristics of assemblages such as energy and nutrient flow (Saint-Germain et al. 2007; Wang et al. 2009), we used it as the metric to describe assemblages functionally (i.e., rather than abundance). We estimated the biomass of each beetle using length:biomass regressions [31, 32], using measured body length or a known average length of common species collected previously [26].

Voucher specimens of all species are now contained in at least one of the following collections: Lyman Entomological Museum (Sainte-Anne-de-Bellevue, Québec, Canada), Canadian National Collection of Insects, Arachnids and Nematodes (Ottawa, Ontario, Canada), Canadian Museum of Nature (Ottawa, Ontario, Canada).

### Environmental variables

We quantitatively assessed the plant community at each location by randomly establishing five 1 m^2^ quadrats in and adjacent to the trap grid in each habitat replicate. To characterize the vegetation in each quadrat we used a % cover classification system, the Braun-Blanquet scale [33]. We assigned plants to a class (forbs, shrubs, graminoids, mosses, lichens), gave each class a Braun-Blanquet score of 1 to 6, and determined the mean score for each class for each location. We measured the maximum height of the vegetation of any class (MaxVegHt) in each quadrat, and determined an average of these heights for each location.

Climate data are available online (Canadian National Climate Data and Information Archive (http://climate.weatheroffice.gc.ca). We used climate normals (calculated using at least 15 years of data taken between 1981–2010) recorded at the weather station closest to each location to obtain the following: mean annual temperature (MeanTemp), mean maximum temperature (MaxTemp), mean minimum temperature (MinTemp), mean temperature of warmest month (WarmMean), mean temperature of coldest month (ColdMean), mean total annual precipitation (TotPrecip), mean degree days above zero (DDA0), mean degree days below zero (DDB0), mean wind speed (Wind), mean annual days with sunshine (SunDays), mean annual total sunshine hours (SunHrs), and number of frost-free days (Frost). Given their proximity, we considered all replicates at the same location to have the same climate conditions.

### Data analyses

We conducted all analyses using R, version 3.1.1 (R Core Team, 2014). We described the total abundance of beetles at each location. While our sampling was standardized, the resulting sample sizes were unequal and there were many rare species. Therefore, in addition to calculating the observed species richness, observed functional group richness, Simpson’s diversity, and Pielou’s evenness, we also used the Chao1 index [34], an asymptotic estimator, to generate unbiased estimates of expected species richness at each location. The analyses were performed using the vegan package [35].

To test whether beetles would conform to predicted latitudinal gradients of diversity, we used linear regressions to test the relationship between each diversity index and latitude. The indices were log-transformed prior to running the models to ensure data met assumptions of normality. We removed extreme outliers to improve model fit: IQA for observed species richness, Chao1 and functional richness, and NOR for evenness and Simpson’s index.

In addition to identifying latitudinal diversity patterns, we wanted to determine if latitude has an indirect effect on diversity that is mediated by other biotic, climatic or spatial factors. Confirmatory path analysis is one method of testing multivariate causal hypotheses that cannot be tested through randomized experiments [36]. We used Shipley’s d-separation test of causal hypotheses [36, 37] to analyse the relationships between latitude, diversity, and mechanistic factors. In this analysis, the causal hypotheses are represented by a set of structural equations that are visualized as a path model (or directed acyclic graph; DAG), and these causal relationships imply independence relations between other pairs of variables (a basis set), either directly or after conditioning on other variables. Using the package ggm [38] we developed three alternative DAGs with hypothesised relationships between latitude, species and functional diversity, and several mechanistic variables, then generated the basis sets of the conditional independencies resulting from the models.

The initial list of mechanisms considered included the climate and vegetation variables described in the previous section. In order to avoid issues associated with autocorrelation and to achieve the most parsimonious DAG, we reduced the number of variables in the model by considering the statistical and ecological relationships between the variables. First we visualized the data with scatterplots of all pairwise combinations of variables. All temperature and sun-related variables were correlated, so MeanTemp was selected as a proxy for all temperature measures. There were some missing Wind values so this variable was omitted. Precipitation was moderately correlated with MeanTemp, but since it captures a very different component of climate, and because moisture can be a constraint for northern arthropods [18, 30], it was retained. All vegetation class Braun-Blanquet scores were retained, as was MaxVegHt. Next, we examined the variance inflation factors using the vif function in the HH package [39]. Some inflation factors were high due to relationships between different vegetation classes, suggesting we should select one representative vegetation variable. We additionally performed stepwise AIC model selection, which selected MeanTemp, MaxVegHt and TotPrecip as independent variables. These steps and results led us to conclude the selection with these three ecologically significant, noncolinear (VIF between 2.2 and 3.5) variables.

**Fig 1:**
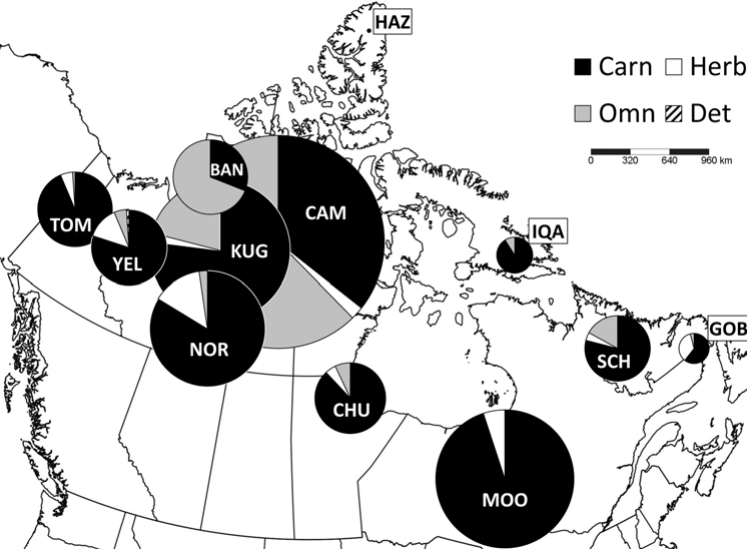
Map of the 12 study locations (North Pole Azimuthal projection), showing the spatial distribution of functional groups. FGs were pooled into trophic groups, and the pie charts show the proportion of the total site biomass represented by each trophic group: carnivore (black), herbivore (white), omnivore (grey) and detritivore (diagonal lines). Pie chart sizes are graduated according to the proportion of the entire study’s beetles collected at that site. High arctic sites: HAZ (Lake Hazen, NU); BAN (Banks Island, NWT); CAM (Cambridge Bay, NU); IQA (Iqaluit, NU). Subarctic sites: KUG (Kugluktuk, NU); TOM (Tombstone Mtns., YT); CHU (Churchill, MB); SCH (Schefferville, QC). North boreal sites: NOR (Norman Wells, NWT); YEL (Yellowknife, NWT); GOB (Goose Bay, NFLD); MOO (Moosonee, ON).

We considered three plausible alternative models to test the relationship between latitude and speices richness (Fig 1A–1C). These included the simplest model (Fig 1A), where only latitude acts on the mediating effects, and two models where interactions between mediating factors were considered. In model B, temperature (T) is directly influenced by latitude, which in turn affects plant height and precipitation. In model C, precipitation additionally influences vegetation height. For each DAG, we tested all hypothesized independences between these variables and then conducted overall tests of the models using Fisher’s C tests. We performed a separate path analysis for functional group richness. Again, we developed three plausible models, using the best-fit DAG for species richness as a starting point (Fig 1, Model 1), because it suggested that interactions between temperature, vegetation and precipitation were not important. Since functional group richness is likely to increase as more species are added (as shown earlier, there is a positive relationship between species and functional richness), we included species richness in the model. The three models we developed were designed to determine whether: a) latitude acts directly on functional richness, b) the effects of latitude are mediated by spatial or biotic variables, and/or c) species richness provides a second level of mediation (i.e., that the effects of temperature, vegetation and precipitation on functional richness are solely or additionally mediated by species richness).

We hypothesized that assemblages would demonstrate spatial (latitudinal) gradients of similarity. To test this, we visualized species and functional assemblages at each location with non-metric multidimensional scaling (NMDS) ordinations, using the rich [40] and vegan [35] packages. Non-metric multidimensional scaling is an indirect ordination approach that maximizes the rank order correlation between distances in a distance matrix, and the function we used (metamds) uses multiple random starting configurations to find a stable global solution for the ordination. Assemblages that are more similar to each other are arranged more closely in the ordination space. In this case, we generated the ordinations using Bray-Curtis distance matrices of log+1 transformed abundances (species) or biomasses (functional groups). Because the species assemblages at HAZ were composed of only a single species, we omitted the location from the NMDS analyses. We were also interested in the relationship between species and functional assemblages, so we compared the congruence of the two ordinations using Procrustes rotation analysis, and assessed the correlation between then using a permutational statistic calculated by the function protest [41].

To test the relationships between assemblage structure and spatial, climatic and biotic factors, the envfit function in the vegan package was used to fit each factor on the ordinations as vectors. The direction of each vector indicates the direction in which the linear change in the variable is the fastest, and the length of the vector is proportional to the strength of the correlation between the variable and the position of the assemblages in ordination space. This function provides an objective interpretation of the results of unconstrained ordination analyses and generates a measure of fit as well as a significance value based on a permutation test (1000 permutations).

## Results

We collected 9062 beetles: 2832 in the high arctic, 3275 in the subarctic and 2955 in the north boreal zone. Abundances varied between locations, ranging from 14 individuals collected from the northernmost location (Lake Hazen, NU), to 1696 individuals from Cambridge Bay, NU, also in the high arctic (Fig 1). There was no relationship between latitude and abundance. Thirty-four families and 155 genera were represented in the collection. Over 80% of the collection was comprised of three families: Carabidae (6221 individuals, 68.8% of total number of specimens), Staphylinidae (870, 9.6%), and Cryptophagidae (247, 2.7%). We found staphylinids at all locations, carabids at all locations with the exception of Lake Hazen, and cryptophagids only from locations in the subarctic and boreal zones. We provide a list of all taxa in S1 Table, and a complete dataset of individual specimen records is available at http://doi.org/10.5886/5dvj8642.

Species-level identifications were done whenever possible, though we focused our efforts on identifying the most abundant and widespread taxa (e.g., Carabidae, Staphylinidae) and those that are taxonomically well known. In total, we identified 464 species and morphospecies, and richness ranged from a single species observed in Lake Hazen, to 115 in the north boreal location Moosonee, ON. Among the samples were 15 new provincial and/or territorial records (denoted by an asterisk, *, next to the species name in S1 Table). Most species were found at three or fewer locations, but others were more widespread. For example, in the high and subarctic zones, *Pterostichus haematopus*, *P*. *brevicornis*, *P*. *caribou* and *Amara alpina* were particularly abundant and widely distributed; together, these four species represented 44.0% of the total catch.

Eighteen functional groups were identified, representing diverse specialist and generalist herbivores and carnivores, omnivores, and saprophages, with different food sources and modes of feeding (S2 Table). Functional group richness ranged from 1 in Lake Hazen to 13 groups in two of the subarctic locations (Norman Wells and Yellowknife, NWT) and one boreal location, Moosonee. Carnivores comprised the majority of the biomass in all sites except Goose Bay, NFLD, which was the only location where herbivores had the greatest proportion of biomass (Fig 1). Strict herbivores were largely absent from the subarctic and high arctic locations, but were well represented in the boreal sites. Although Moosonee had very high species and functional richness, high numbers of two very large predacious carabid beetles were caught (*Carabus maeander* and *C*. *melanarius*), and these dominated the overall biomass (Fig 1). Omnivores were exceptionally prominent in the high arctic, represented primarily by the opportunistically predacious granivore, *Amara alpina* (Fig 1).

### Diversity

Both observed species richness (P = 0.002, R^2^ = 0.6345, F = 18.36, df = 9) and expected species richness (P = 0.002, R^2^ = 0.6411, F = 18.86, df = 9) exhibited strong negative relationships with latitude. The Simpson’s index similarly declined significantly with latitude (P = 0.0043, R^2^ = 0.5723, F = 14.38, df = 9), but evenness had no significant spatial pattern (P = 0.06, R^2^ = 0.3078, F = 5.002, df = 8). Functional group richness had a highly significant negative relationships with latitude (P = 0.0007, R^2^ = 0.7101, F = 25.49, df = 9). Functional group richness and species richness have a very strong positive linear relationship (P < 0.0001, R^2^ = 0.9307, F = 148.6, df = 10).

**Fig 2:**
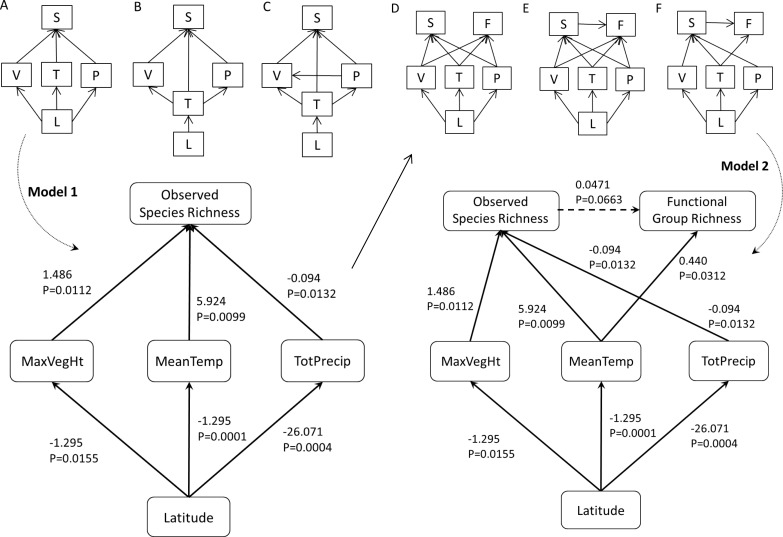
Three alternative (A, B, and C) directed acyclic graphs (DAG) of hypothesized direct and indirect effects on species richness (S). Model 1: results of best fit path model (derived from model A). D, E and F are alternative DAGs of hypothesized effects on functional group richness (F). Model: results of the best fit path model (from model F). The direction of the arrow indicates the direction of the relationship. Solid lines indicate a significant relationship. Estimated coefficients and P-values are shown for each relationship. Latitude (L), TotPrecip (P), MeanTemp (T) and MaxVegHt (V)

In our path analysis of species diversity, we opted to include MeanTemp, TotPrecip, and MaxVegHt, as mediating effects. Fisher’s C tests (which simultaneously test all independencies in a DAG) indicated that, while all three models were good fits with no important dependencies missing, the first and simplest model (Fig 2A) provided the best outcome (Fishers’s C = 3.637, P = 0.888). The path analysis indicates that species richness is indirectly affected by latitude. MeanTemp, MaxVegHt and TotPrecip all decline as latitude increases. MeanTemp and MaxVegHt have positive effect on species richness, while TotPrecip has a negative effect. The strength and direction of the relationships determined by the path analysis for the best-fit model are shown in Fig 2 (Model 1). For functional diversity, Fisher’s C tests indicated that all three models (D-F) were acceptable, but model F was the best fit (Fisher’s C = 5.099, P = 0.984). In this path analysis we found that MeanTemp and species richness both had a positive effect on functional richness, and that latitude had an indirect negative effect through these mediating factors (Fig 2, Model 2). Relationships between functional group richness, and MaxVegHt and TotPrecip, did not improve the model.

### Assemblage structure

**Fig 3:**
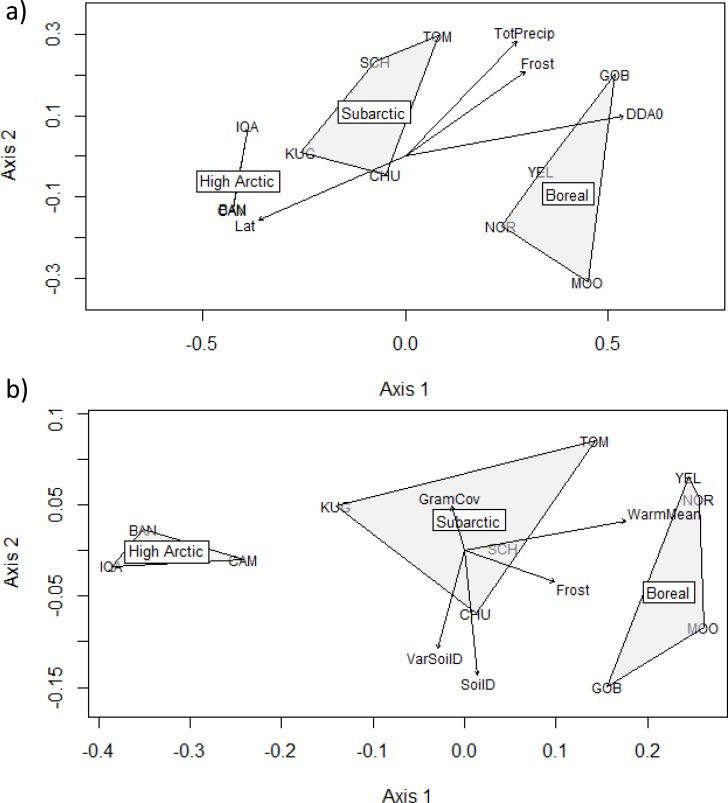
Non-metric multidimensional scaling ordinations showing similarities between a) species, and b) functional assemblages from at each location. Shaded polygons are used to delimit the ordination space occupied by sites contained within the same ecoclimatic zone. Spatial, climatic, and biotic variables that are significantly correlated with the assemblages are plotted on the ordination space as vectors (for clarity, non-significant variables are omitted, and only the most important temperature-related variable is included for each ordination); the length of the vector indicates the strength of the correlation. (HAZ has been omitted as an outler from both ordinations, as it contains only one, uncommon species).

Solutions were reached for the NMDS ordinations of the species assemblages (stress = 0.079913, Fig 3a) and the functional assemblages (stress = 0.05501, Fig 3b). With the exception of CAM and BAN, whose species assemblages essentially overlap in the ordination space, all locations displayed distinct species and functional assemblages. The arrangement of the locations in the ordinations indicated that those in the same ecoclimatic zone had similar assemblages, and that there were clear delimitations between zones. Functional assemblages from the western and eastern part of the continent show a slight tendency to assemble closer together, even between ecoclimatic zones; this is more pronounced for locations in the north boreal and subarctic. A gradient of similarity was evident between ecoclimatic zones: assemblages in the subarctic were more like those found in the high arctic, while north boreal assemblages were more similar to subarctic than to high arctic assemblages. Species assemblage structure was significantly correlated with latitude (S3 Table). According to the Procrustes rotation analysis, the species and functional ordinations were correlated (Procrustes sum of squares = 0.36157, Procrustes correlation, r = 0.7964, P = 0.001, 999 permutations).

Species and functional assemblage structure were significantly correlated with all temperature-driven climatic factors, with the exception that functional assemblages were not related to DDBO (S3 Table). Both assemblage types were also significantly related to Frost. Species assemblage structure was additionally significantly related to TotPrecip, while functional assemblage also has significant relationships with SoilD, VarSoilD and GramCov. Otherwise, the plant community composition is not related to either species or functional assemblage structure. Variables that are significantly related to assemblage structure are plotted as vectors in Fig 3 (only the temperature factor with the strongest relationship to assemblage structure is shown, for clarity).

## Discussion

At a large spatial extent, the diversity and assemblage structure of northern beetles show strong latitudinal gradients, primarily due to mediating effects of climate, particularly temperature. Our research spanned a latitudinal gradient of 30°, included three ecoclimatic zones, and used standardized field sampling of terrestrial beetles to uncover biodiversity patterns across a large geographic extent. Macroecological studies with extents that range less than 20° of latitude are unlikely to show clear spatial patterns of species richness, even if such patterns exist [3]; our findings should therefore provide an accurate portrayal of spatial biodiversity patterns in the far north. We found that beetle species richness (observed, predicted, and as measured by the Simpson’s index) exhibits classical negative relationships with latitude. This aligns with general observations of beetle richness drawn from presence/absence data in arctic and northern boreal regions [19] and with similar patterns described for other insect taxa at a continental scale in temperate regions, including ants in Europe, and grasshoppers, butterflies and dung beetles in North America [3].

Evenness showed a tendency to decline with increasing latitude, but the gradient was non-significant. Species richness and evenness can be, and often are, similar along latitudinal gradients, but they may also show no or even negative relationships with each other, as they may reflect different aspects of spatial variation in species assemblages and different responses to ecological factors [42, 43]. Alternatively, if assemblages are more species-rich because they possess greater numbers of rare species, then we might expect latitudinal decreases in richness to be accompanied by greater evenness [3]. While the majority of our rare species (singletons and doubletons) were indeed located in the more southerly locations, this did not translate to a positive latitudinal evenness trend.

In our study, functional group richness showed a very strong classical spatial gradient, decreasing sharply with increasing latitude. Studies on large-scale latitudinal patterns of animal functional diversity are scarce and their conclusions are variable. Stevens et al. [14], for example, found that New World bat functional diversity, richness and dominance strongly increased towards the equator, and mammalian functional diversity generally appears to display the same pattern [15]. Conversely, an analysis of invertebrates collected from 1000 stream monitoring stations in the US showed that functional richness decreased only weakly with increased latitude [44].

Specific functional groups or guilds may display different latitudinal diversity patterns. For example, a large survey of the freshwater detriviorous shredder guild revealed much higher diversity at higher latitudes [8]. Similarly, among several functional groups of syrphid flies sampled across Europe, only saphrophage diversity was significantly related to latitude [45], and this was also a positive relationship. Some evidence exists that trophic levels of beetles (i.e., carnivores, herbivores and saprophages) display latitudinal gradients. Examinations of selected groups of carnivorous and herbivorous beetles from island systems suggest that carnivores account for smaller proportions of the fauna in the southern hemisphere, increasing through the tropics into the far north, while the proportion of herbivores declines from northern latitudes to the equator, with little change further south [46]. Gaston’s [46] meta-analysis of carnivore:non-carnivore ratios in beetle fauna similarly found more predatory species in samples from northern temperate regions than from the tropics.

We found no significant relationship between latitude and abundance. However, abundance was especially low in the most extreme northern location, and generally higher further south (with notable exceptions), which aligns well with Danks’ observation that in North America, beetles contribute fewer species to the overall pool of insect fauna at higher latitudes compared to mid-latitudes [47]. Gaston has suggested that an increase in non-beetle insects at higher latitudes may translate into an increase in food energy contained in the non-beetle part of the community, meaning there could be more potential energy available to predatory beetles (i.e., more non-beetles on which to prey) [46]. This might explain, in part, the greater proportion of carnivorous and omnivorous beetles collected in our study in high and subarctic locations (i.e., compared to boreal locations).

Ecoclimatic zones are defined by a variety of ecological characteristics, including climate, soil, humidity and vegetation communities [28]. Since beetle assemblages were most alike when contained within the same ecoclimatic zone, it stands to reason that assemblages are highly dependent on at least some of these characteristics. A number of recent studies suggest that temperature [26, 48–50] and plant community composition [51, 52] are associated with temporal and spatial changes in arctic arthropod assemblage structure at the local or regional level. Northern insect activity levels can also be locally influenced by temperature [30, 53] or wind [54].

Although the primary “causes” of the large scale relationships between biodiversity and latitude remain under dispute, there is nevertheless good evidence that they are climatic rather than biotic (i.e., involving species interactions), whether modern or on an evolutionary time scale [3, 7, 55]. We provide support for this with evidence that latitudinal patterns of beetle species and functional richness are mediated by mean annual temperature and total annual precipitation. We also demonstrate that beetle assemblage structure is strongly associated with multiple metrics of temperature minima and maxima, lending support to the idea that climatic gradients are key drivers of large-scale species assembly and ecological function.

The assemblage structure of northern beetles is linked at least in part to the depth of the active soil layer and the height of the surrounding vegetation, while functional assemblage structure is also related to the presence of graminoids; similar patterns have been observed for other macroarthropod assemblages [56, 57]. These associations are conceivably due to correlations between temperature, and soil depth and plant height. For example, experimental warming of plants by 1–3°C has been shown to significantly increase the height of shrubs and graminoids in the arctic tundra in less than two years [58]. Increased air temperature is well known to affect increases in soil temperature and active layer depth, and to reduce permafrost stability in the Arctic [59–61]. This may be additional support for climate (temperature) as a key determinant of terrestrial insect assemblage structure at large spatial scales.

In this study, we show that latitudinal gradients of species richness are correlated with those of functional richness. Additionally, we find that continental-scale patterns of functional assemblage structure are correlated with those of species assemblages, and that functional assemblages reflect climatic gradients just as strongly and in a near-identical manner as species assemblages. We suggest that climate change is likely to have significant effects on both the structure and function of ecological assemblages, and that function-driven examinations of assemblages are just as ecologically meaningful and informative as those using a traditional, species-level approach if the aim is to identify or track changes in biodiversity and assemblage composition over time or space.

The use of functional groups and functional diversity as complementary [62] or alternative metrics of biodiversity (“biodiversity surrogacy”) has been supported by many authors for a variety of taxa and ecosystems [63, 64, 65]. There are multiple lines of evidence that indicate very strong ties between functional and taxonomic diversity [66] and that species-level responses to environmental changes or gradients are not lost at higher taxonomic resolutions [67]. From a practical perspective (e.g., in the context of ecological monitoring programs) most taxa can be assigned to a functional group or guild after being identified to subfamily, even family and occasionally order; a much more feasible undertaking for non-specialist workers, or volunteers.

## Conclusions

Our data have provided a valuable test of macroecological diversity patterns and their underlying processes across the three northernmost ecoclimatic zones of North America. We collected over 9,000 beetles from diverse taxonomic and functional groups, and demonstrated that beetles conform to classical latitudinal gradients of diversity. Our data reveal a clear relationship between taxonomic and functional assemblage structure, both of which are strongly associated with latitude. Although climate appears to be a likely candidate for the key mechanism underlying these patterns, further field-based assessments are required.

To our knowledge, no other study has presented a quantitative examination of spatial patterns in species or functional assemblage structure across a spatial extent as large as the one we have presented here, using standardized field samples (i.e., including abundance or density). Although it seems intuitive that, if species-level spatial patterns exist, then assemblage-level patterns should similarly be displayed, it is nevertheless difficult to say whether the assemblage-level spatial patterns we have described here can be generalized to other systems, or whether they are specific to northern regions or biomes with extreme climates. We suggest that more effort must be made to assess not only the number of species present in ecosystems over large spatial scales, but also their relative contributions to the structure and functioning of their assemblages.

Northern terrestrial diversity is dominated by a rich and unique arthropod fauna. Terrestrial insects perform many critical ecological functions in northern biomes, including plant pollination, decomposition, and provision of food for highly valued vertebrates [68]. Their richness and ability to occupy a wide variety of functional ecological niches present significant opportunities for biodiversity research and conservation efforts. The imminent threats of climate change in the fragile and susceptible regions of the Arctic [69] have prompted the conception of several international terrestrial biodiversity monitoring initiatives, such as [68]. Beetles are highly sensitive to changes in climate and, as we have shown, reflect these changes in their diversity, distribution, and assemblage structure, making them ideal taxa for targeted long-term diversity monitoring.

## Supporting Information

S1 Table Diversity and abundance of beetle species found at each location, listed in alphabetical order by Family. Asterisk denotes new provincial or territorial record.
(DOCX)

S2 Table Relative proportion of total beetle biomass from each of the 12 sampling locations, in each functional group. Molluscivores, Collembola and Mite Specialist Predators, Generalist Omnivores, Non-feeding Adults, Xylophages, and Micropolyvores have been omitted for clarity because their contributions to the biomass are extremely small (< 1% of total) in all locations.
(DOCX)

S3 Table Relationships between climatic and environmental factors, and the NMDS ordinations of species (left) and functional (right) assemblages found at the twelve sites. R-squared and significance values are determined using a permutational approach that fits each driver on the ordination space as a vector.
(DOCX)
