# Supplementary material for: Drivers and Patterns of Ground-Dwelling Beetle Biodiversity across Northern Canada
Source: PLoS One. 2015 Apr 22;10(4):e0122163. doi: 10.1371/journal.pone.0122163 (PMC4406721; doi:10.1371/journal.pone.0122163)
Supplement: S1 Table — Asterisk denotes new provincial or territorial record. (DOCX) [file pone.0122163.s001.docx]

|  |  | |  | **High Arctic** | | | | **Subarctic** | | | | **North Boreal** | | | |
| --- | --- | --- | --- | --- | --- | --- | --- | --- | --- | --- | --- | --- | --- | --- | --- |
| **Family**  **Subfamily** | | | **Species** | **HAZ** | **BAN** | **CAM** | **IQU** | **KUG** | **TOM** | **CHU** | **SCH** | **NOR** | **YEL** | **GOB** | **MOO** |
| Anthicidae | | |  |  |  |  |  |  |  |  |  |  |  |  |  |
|  | Anthicinae | |  |  |  |  |  |  |  |  |  |  |  |  |  |
|  |  | | *Anthicus* sp.1 |  |  |  |  |  |  |  |  | 16 |  |  |  |
| Buprestidae | | |  |  |  |  |  |  |  |  |  |  |  |  |  |
|  |  | | *Buprestinae* |  |  |  |  |  |  |  |  |  |  |  |  |
|  |  | | *Anthaxia* (*Melanthaxia*) *inornata* |  |  |  |  |  |  |  |  | 90 | 16 |  | 7 |
| Byrrhidae | | |  |  |  |  |  |  |  |  |  |  |  |  |  |
|  | Byrrhinae | |  |  |  |  |  |  |  |  |  |  |  |  |  |
|  |  | | *Byrrhus* sp. |  |  |  |  |  |  | 1 |  |  |  |  |  |
|  |  | | *Byrrhus* sp. 2 |  |  |  |  |  |  |  |  |  | 3 |  |  |
|  |  | | *Byrrhus* sp. 3 |  |  |  |  |  |  |  | 3 |  |  |  |  |
|  |  | | *Byrrhus* sp. 4 |  |  |  |  | 3 |  |  |  |  |  |  |  |
|  |  | | *Byrrhus* sp. 5 |  |  |  |  |  |  |  |  | 1 |  |  |  |
|  |  | | *Byrrhus* sp. 6 |  |  |  |  |  | 19 |  |  |  |  |  |  |
|  |  | | *Byrrhus* sp. 7 |  |  |  |  |  |  |  |  |  |  |  | 37 |
|  |  | | *Byrrhus* sp. 8 |  |  |  |  |  | 2 |  |  |  |  |  |  |
|  |  | | *Cytilus sp.* |  |  |  |  |  |  |  |  | 4 | 3 |  | 13 |
|  |  | | *Simplocara* sp. |  |  |  |  |  |  | 1 |  |  |  |  |  |
|  | Syncalyptinae | |  |  |  |  |  |  |  |  |  |  |  |  |  |
|  |  | | *Curimopsis setulosa* |  |  |  |  |  |  |  |  |  | 1 |  |  |
|  |  | | *Curimopsis* sp. |  |  |  |  |  |  |  | 1 |  |  |  |  |
|  |  | | *Curimopsis* sp. 2 |  |  |  |  |  | 3 |  |  |  |  |  |  |
| Cantharidae | | |  |  |  |  |  |  |  |  |  |  |  |  |  |
|  | Cantharinae | |  |  |  |  |  |  |  |  |  |  |  |  |  |
|  |  | | *Dichelotarsus laevicollis* |  |  |  |  |  |  |  | 1 |  |  |  |  |
|  |  | | *Dichelotarsus puberulus* |  |  |  |  |  |  |  | 6 |  |  |  | 1 |
|  |  | | *Dichelotarsus* sp. |  |  |  |  |  |  | 29 |  |  |  |  |  |
|  |  | | *Rhagonycha fraxini* |  |  |  |  |  |  |  |  |  |  | 28* | 1 |
|  |  | | *Rhagonycha mandibularis* |  |  |  |  |  |  | 87 |  |  |  |  |  |
|  |  | | *Rhagonycha* sp. |  |  |  |  | 2 |  |  |  |  |  |  |  |
| Carabidae | | |  |  |  |  |  |  |  |  |  |  |  |  |  |
|  | | Carabinae |  |  |  |  |  |  |  |  |  |  |  |  |  |
|  | |  | *Carabus chamissonis* |  |  |  |  | 1 |  | 48 |  | 42 |  |  |  |
|  | |  | *Carabus granulatus* |  |  |  |  |  |  |  |  |  |  |  | 174 |
|  | |  | *Carabus maeander* |  |  |  |  |  |  |  |  | 30 |  |  | 6 |
|  | |  | *Carabus serratus* |  |  |  |  |  |  |  |  |  |  | 27* |  |
|  | |  | *Carabus taedatus* |  |  |  |  |  |  | 24 |  |  |  |  |  |
|  | |  | *Carabus truncaticollis* |  |  |  |  |  | 100 |  |  |  |  |  |  |
|  | |  | *Carabus vietinghoffii* |  |  |  |  | 6 |  |  |  | 8 |  |  |  |
|  | |  | *Scaphinotus bilobus* |  |  |  |  |  |  |  |  |  |  |  | 1 |
|  | |  | *Sphaeroderus stenostomus* |  |  |  |  |  |  |  |  |  |  |  | 5 |
|  | | Cicindelinae |  |  |  |  |  |  |  |  |  |  |  |  |  |
|  | |  | *Cicindela limbalis* |  |  |  |  |  |  |  |  | 2 |  |  |  |
|  | |  | *Cicindela longilabris longilabris* |  |  |  |  |  |  |  |  | 2 |  |  |  |
|  | | Elaphrinae |  |  |  |  |  |  |  |  |  |  |  |  |  |
|  | |  | *Blethisa catenaria* |  |  |  |  | 9 |  |  |  |  |  |  |  |
|  | |  | *Blethisa quadricollis* |  |  |  |  |  |  |  |  |  |  |  | 2 |
|  | |  | *Diacheila arctica* |  |  |  |  |  |  |  |  | 1 |  |  |  |
|  | |  | *Diacheila polita* |  |  |  |  |  | 16 |  |  |  |  |  |  |
|  | |  | *Elaphrus clairvillei* |  |  |  |  |  |  | 6 |  | 4 |  |  | 4 |
|  | |  | *Elaphrus fuliginosus* |  |  |  |  |  |  |  |  |  | 1* |  |  |
|  | |  | *Elaphrus lapponicus* |  |  |  |  |  | 27 | 16 |  | 9 | 4 |  |  |
|  | |  | *Elaphrus lecontei* |  |  |  |  |  |  |  |  |  |  |  | 1 |
|  | | Harpalinae |  |  |  |  |  |  |  |  |  |  |  |  |  |
|  | |  | *Agonum affine* |  |  |  |  |  |  | 9 |  | 11 |  |  | 7 |
|  | |  | *Agonum consimile* |  |  |  |  |  |  |  |  | 1 |  |  |  |
|  | |  | *Agonum exaratum* |  |  | 2 |  |  |  |  |  |  |  |  |  |
|  | |  | *Agonum gratiosum* |  |  |  |  |  |  | 3 |  | 29 | 100 | 1 | 23 |
|  | |  | *Agonum metallescens* |  |  |  |  |  |  |  |  |  |  |  | 8 |
|  | |  | *Agonum mutatum* |  |  |  |  |  |  |  |  |  |  | 2 |  |
|  | |  | *Agonum quinquepunctatum* |  |  |  |  |  |  | 6 |  | 6 |  |  |  |
|  | |  | *Agonum superioris* |  |  |  |  |  |  |  |  |  |  |  | 2 |
|  | |  | *Agonum thoreyi* |  |  |  |  |  |  |  |  |  |  |  | 1 |
|  | |  | *Amara alpina* |  | 303 | 804 | 111 | 213 |  | 28 | 3 |  |  |  |  |
|  | |  | *Amara erratica* |  |  |  |  |  |  |  |  | 1 |  |  |  |
|  | |  | *Amara hyperborea* |  |  |  |  |  |  |  | 83 | 4 | 6 |  |  |
|  | |  | *Amara laevipennis* |  |  |  |  |  |  | 1 |  |  | 4 | 21* |  |
|  | |  | *Amara littoralis* |  |  |  |  |  |  |  |  |  |  |  | 1 |
|  | |  | *Amara pseudobrunnea* |  |  |  |  |  | 1 |  | 10 | 1* | 2 | 1 |  |
|  | |  | *Amara torrida* |  |  |  |  |  |  |  |  | 1 |  |  | 1 |
|  | |  | *Badister obtusus* |  |  |  |  |  |  |  |  | 1 | 2 |  | 1 |
|  | |  | *Bradycellus neglectus* |  |  |  |  |  |  |  |  |  | 2* |  |  |
|  | |  | *Bradycellus nigrinus* |  |  |  |  |  |  |  |  |  |  |  | 4 |
|  | |  | *Calathus ingratus* |  |  |  |  |  |  |  | 2 | 9 | 10 | 9 | 3 |
|  | |  | *Chlaenius alternatus* |  |  |  |  |  |  |  |  | 38 | 2 |  |  |
|  | |  | *Chlaenius lithophilus* |  |  |  |  |  |  |  |  |  |  |  | 1 |
|  | |  | *Chlaenius niger* |  |  |  |  |  |  |  |  |  |  |  | 1 |
|  | |  | *Cymindis unicolor* |  |  |  |  | 2 | 1 | 1 |  |  |  |  |  |
|  | |  | *Dicheirotrichus cognatus* |  |  |  |  |  |  |  |  |  |  |  | 5 |
|  | |  | *Harpalus solitaris* |  |  |  |  |  |  |  |  | 1 | 1 |  |  |
|  | |  | *Harpalus nigritarsis* |  |  |  |  |  | 6 |  |  | 9 | 3 |  |  |
|  | |  | *Harpalus pleuritieus* |  |  |  |  |  |  |  |  | 1 |  |  |  |
|  | |  | *Platynus decentis* |  |  |  |  |  |  |  |  |  |  |  | 1 |
|  | |  | *Platynus mannerheimii* |  |  |  |  |  |  |  | 1 |  |  |  | 2 |
|  | |  | *Poecilus lucublandis* |  |  |  |  |  |  |  |  |  |  |  | 6 |
|  | |  | *Pterostichus adstrictus* |  |  |  |  |  |  |  |  | 20 | 4 |  | 15 |
|  | |  | *Pterostichus arcticola* |  |  |  |  |  | 18 | 1 | 2 |  |  |  |  |
|  | |  | *Pterostichus barryorum* |  |  |  |  | 10 |  |  |  |  |  |  |  |
|  | |  | *Pterostichus brevicornis* |  |  | 2 |  | 392 | 64 | 80 | 3 |  |  |  |  |
|  | |  | *Pterostichus caribou* |  | 154 | 846 |  | 325 |  | 69 |  |  |  |  |  |
|  | |  | *Pterostichus haematopus* |  |  |  | 306 | 106 | 6 | 54 | 110 | 3 | 1 |  |  |
|  | |  | *Pterostichus hudsonicus* |  |  |  |  | 4 |  |  |  |  |  |  |  |
|  | |  | *Pterostichus melanarius* |  |  |  |  |  |  |  |  |  |  |  | 204 |
|  | |  | *Pterostichus parasimilis* |  |  |  |  |  | 244 |  |  |  |  |  |  |
|  | |  | *Pterostichus patruelis* |  |  |  |  |  |  |  |  | 6* | 2* | 1 | 9 |
|  | |  | *Pterostichus pensylvanicus* |  |  |  |  |  |  |  |  | 19 |  | 6* |  |
|  | |  | *Pterostichus pinguedineus* |  |  |  |  |  |  |  | 1 |  |  |  |  |
|  | |  | *Pterostichus punctatissimus* |  |  |  |  |  |  | 71 |  | 18 | 22 |  |  |
|  | |  | *Pterostichus tareumiut* |  | 197 |  |  |  |  |  |  |  |  |  |  |
|  | |  | *Pterostichus tenuis* |  |  |  |  |  |  |  |  |  |  |  | 4 |
|  | |  | *Pterostichus vermiculosus* |  |  | 1 |  | 42 |  |  |  |  |  |  |  |
|  | |  | *Syntomus americanus* |  |  |  |  |  |  |  |  |  | 2 | 1 |  |
|  | |  | *Synuchus impunctatus* |  |  |  |  |  |  |  |  |  |  |  | 1 |
|  | | Loricerinae |  |  |  |  |  |  |  |  |  |  |  |  |  |
|  | |  | *Loricera pilicornis* |  |  |  |  |  |  | 1 |  |  |  |  |  |
|  | | Nebriinae |  |  |  |  |  |  |  |  |  |  |  |  |  |
|  | |  | *Nebria gyllenhali* |  |  |  |  |  |  |  | 3 |  |  |  |  |
|  | |  | *Notiophilus aquaticus* |  |  |  |  |  |  |  | 1 |  |  |  |  |
|  | |  | *Notiophilus borealis* |  |  |  |  | 22 | 5 | 9 | 3 |  |  |  |  |
|  | |  | *Notiophilus intermedius* |  |  |  |  |  |  |  |  | 1* | 1* |  |  |
|  | |  | *Notiophilus semistriatus* |  |  |  |  |  |  |  |  |  | 2* | 1 |  |
|  | | Patrobinae |  |  |  |  |  |  |  |  |  |  |  |  |  |
|  | |  | *Patrobus foveocollis* |  |  |  |  |  |  |  | 3 |  | 1 |  |  |
|  | |  | *Patrobus longicornis* |  |  |  |  |  |  |  |  |  |  |  | 5 |
|  | |  | *Patrobus septentrionis* |  |  |  |  |  |  |  | 3 | 10 |  |  |  |
|  | |  | *Patrobus stygicus* |  |  |  |  |  |  |  |  | 3 |  |  |  |
|  | | Scaritinae |  |  |  |  |  |  |  |  |  |  |  |  |  |
|  | |  | *Clivina fossor* |  |  |  |  |  |  |  |  |  |  |  | 5 |
|  | |  | *Dyschirius frigidus* |  |  |  |  |  | 1 |  |  |  |  |  |  |
|  | |  | *Dyschirius globulosus* |  |  |  |  |  |  |  |  |  | 1* |  |  |
|  | |  | *Dyschirius hiemalis* |  |  |  |  |  |  | 13 | 1 | 3 | 15 |  |  |
|  | |  | *Dyschirius integer* |  |  |  |  |  |  |  |  |  |  |  | 18 |
|  | |  | *Dyschirius melanocholicus* |  |  |  |  | 2 | 16 |  |  |  |  |  |  |
|  | |  | *Dyschirius nigricornis* |  |  |  |  |  |  | 6 |  |  |  |  |  |
|  | |  | *Dyschirius subarcticus* |  |  |  |  |  | 1 |  |  |  |  |  |  |
|  | | Trechinae |  |  |  |  |  |  |  |  |  |  |  |  |  |
|  | |  | *Bembidion bimaculatum* |  |  |  |  |  |  |  |  |  |  |  | 2 |
|  | |  | *Bembidion dilaticolle* |  |  |  |  |  |  |  |  |  |  |  | 1 |
|  | |  | *Bembidion diligens* |  |  |  |  |  |  |  |  | 2* |  |  |  |
|  | |  | *Bembidion forestriatum* |  |  |  |  |  |  |  |  |  | 1 |  |  |
|  | |  | *Bembidion fortestriatum* |  |  |  |  |  |  |  |  |  |  |  | 8 |
|  | |  | *Bembidion grapei* |  |  |  |  |  |  |  | 1 |  |  |  |  |
|  | |  | *Bembidion morulum* |  |  |  |  |  |  | 1 |  | 38 | 17 |  |  |
|  | |  | *Bembidion quadratulum* |  |  |  |  |  |  |  |  |  |  | 1* |  |
|  | |  | *Bembidion transparens* |  |  |  |  |  |  |  |  | 1 |  |  | 21 |
|  | |  | *Bembidion versicolor* |  |  |  |  |  |  |  |  |  |  |  | 15 |
|  | |  | *Syntomus americanus* |  |  |  |  |  |  |  |  | 3 |  |  |  |
|  | |  | *Trechus crassiscapus* |  |  |  |  |  |  |  | 1 |  |  |  |  |
| Cerambycidae | | |  |  |  |  |  |  |  |  |  |  |  |  |  |
|  | Lepturinae | |  |  |  |  |  |  |  |  |  |  |  |  |  |
|  |  | | *Pidonia scripta* |  |  |  |  |  |  |  |  |  | 1* |  |  |
|  |  | | *Rhagium inquisitor* |  |  |  |  |  |  |  |  |  |  | 6 |  |
| Chrysomelidae | | |  |  |  |  |  |  |  |  |  |  |  |  |  |
|  | Galerucinae | |  |  |  |  |  |  |  |  |  |  |  |  |  |
|  |  | | *Altica* sp. 1 |  |  |  |  |  |  |  |  | 1 |  |  |  |
|  |  | | *Altica* sp. 2 |  |  |  |  |  |  |  |  |  |  |  | 13 |
|  |  | | *Altica* sp. 3 |  |  |  |  |  |  |  |  |  | 1 |  |  |
|  |  | | *Altica* sp. 4 |  |  |  |  |  |  |  | 1 |  |  |  |  |
|  |  | | *Altica* sp. 5 |  |  |  |  |  |  |  |  |  |  | 2 |  |
|  |  | | *Alticine* sp. |  |  |  |  |  |  | 1 |  |  |  |  |  |
|  |  | | *Alticine* sp.2 |  |  |  |  |  |  |  |  |  |  |  | 1 |
|  |  | | *Alticine* sp. 3 |  |  |  |  |  |  |  |  |  |  |  | 1 |
|  |  | | *Alticine* sp. 4 |  |  |  |  |  |  |  | 20 |  |  |  |  |
|  |  | | *Chaetocnema* sp. |  |  |  |  |  |  |  |  |  |  |  | 1 |
|  |  | | *Chaetocnema* sp.2 |  |  |  |  |  |  |  |  | 2 |  |  |  |
|  |  | | *Crepidodera* sp. |  |  |  |  |  |  |  |  |  |  |  | 8 |
|  |  | | *Glyptina* sp. |  |  |  |  |  |  |  |  | 1 |  |  |  |
|  |  | | *Mantura* sp. |  |  |  |  |  |  |  |  | 1 |  |  |  |
|  |  | | *Neogalerucella pusilla* |  |  |  |  |  |  | 1 |  |  |  |  | 4 |
|  |  | | *Tricholochmaea sp.* |  |  |  |  |  |  |  |  | 3 |  |  | 6 |
|  |  | | *Tricholochmaea vaccinii* |  |  |  |  |  |  |  | 2 |  |  | 3* |  |
| Cleridae | | |  |  |  |  |  |  |  |  |  |  |  |  |  |
|  | Clerinae | |  |  |  |  |  |  |  |  |  |  |  |  |  |
|  |  | | *Trichodes ornatus* |  |  |  |  |  |  |  |  | 40 |  |  |  |
| Coccinellidae | | |  |  |  |  |  |  |  |  |  |  |  |  |  |
|  | Coccinellinae | |  |  |  |  |  |  |  |  |  |  |  |  |  |
|  |  | | *Anisosticta bitriangularis* |  |  |  |  |  |  |  |  | 1 |  |  | 8 |
|  |  | | *Ceratomegilla ulkei* |  |  |  |  |  | 5 | 2 |  |  |  |  |  |
|  |  | | *Coccinella hieroglyphica* |  |  |  |  |  |  |  |  | 1 |  |  |  |
|  |  | | *Hippodamia* sp. |  |  |  |  |  |  |  | 2 |  |  |  |  |
|  |  | | *Hippodamia* sp. 2 |  |  |  |  |  |  |  |  |  | 1 |  |  |
|  |  | | *Coccinellid* sp. |  |  |  |  |  |  |  |  | 1 | 1 |  |  |
|  |  | | *Coccinellid* sp. 2 |  |  |  |  |  |  |  |  |  |  | 4 |  |
|  |  | | *Coccinellid* sp. 3 |  |  |  |  |  |  |  |  | 2 |  |  |  |
|  |  | | *Coccinellid* sp. 4 |  |  |  |  |  |  |  |  | 1 |  |  |  |
|  |  | | *Coccinellid* sp. 5 |  |  |  |  | 5 |  |  |  |  |  |  |  |
|  |  | | *Coccinellid* sp. 6 |  |  |  |  |  |  |  |  | 4 |  |  |  |
|  |  | | *Coccinellid* sp. 7 |  |  |  |  |  |  |  |  | 1 |  |  |  |
|  |  | | *Coccinellid* sp. 8 |  |  |  |  |  |  |  |  |  | 18 |  |  |
|  |  | | *Coccinellid* sp. 9 |  |  |  |  |  |  |  |  |  | 2 |  |  |
|  |  | | *Coccinellid* sp. 10 |  |  |  |  |  | 1 |  |  |  |  |  |  |
|  |  | | *Coccinellid* sp. 11 |  |  |  |  |  |  | 6 |  |  |  |  |  |
| Corylophidae | | |  |  |  |  |  |  |  |  |  |  |  |  |  |
|  |  | | *Corylophid* sp. |  |  |  |  |  |  |  |  |  | 19 |  | 6 |
|  |  | | *Corylophid* sp. 2 |  |  |  |  |  |  |  |  |  | 1 |  | 1 |
|  |  | | *Corylophid* sp. 3 |  |  |  |  |  |  |  |  |  | 1 |  | 1 |
|  |  | | *Corylophid* sp. 4 |  |  |  |  |  |  |  |  |  | 1 |  | 1 |
| Cryptophagidae | | |  |  |  |  |  |  |  |  |  |  |  |  |  |
|  | Atomariinae | |  |  |  |  |  |  |  |  |  |  |  |  |  |
|  |  | | *Atomaria* sp. |  |  |  |  |  |  |  |  |  |  |  | 3 |
|  |  | | *Atomaria* sp. 2 |  |  |  |  |  |  |  |  |  | 1 |  | 18 |
|  |  | | *Atomaria* sp. 3 |  |  |  |  |  |  |  |  |  |  |  | 75 |
|  |  | | *Atomaria* sp. 4 |  |  |  |  |  | 1 |  |  |  |  |  |  |
|  |  | | *Atomaria* sp. 5 |  |  |  |  |  |  |  |  |  | 1 |  |  |
|  |  | | *Tisactia* sp. |  |  |  |  |  |  |  |  | 1 |  |  |  |
|  | Cryptophaginae | |  |  |  |  |  |  |  |  |  |  |  |  |  |
|  |  | | *Cryptophagus* sp. |  |  |  |  |  |  | 4 |  |  |  |  |  |
|  |  | | *Cryptophagus* sp. 2 |  |  |  |  |  |  |  |  | 55 |  |  |  |
|  |  | | *Cryptophagus* sp. 3 |  |  |  |  |  | 2 |  |  |  |  |  |  |
|  |  | | *Cryptophagus* sp. 4 |  |  |  |  |  |  |  |  |  | 31 |  |  |
|  |  | | *Cryptophagus* sp. 5 |  |  |  |  |  |  |  |  | 1 |  |  |  |
|  |  | | *Cryptophagus* sp. 6 |  |  |  |  |  |  |  |  |  |  | 45 |  |
|  |  | | *Cryptophagus* sp. 7 |  |  |  |  |  |  |  | 9 |  |  |  |  |
| Curculionidae | | |  |  |  |  |  |  |  |  |  |  |  |  |  |
|  | Cossoninae | |  |  |  |  |  |  |  |  |  |  |  |  |  |
|  |  | | *Rhyncolus brunneus* |  |  |  |  |  |  |  |  |  | 2 |  |  |
|  | Curculioninae | |  |  |  |  |  |  |  |  |  |  |  |  |  |
|  |  | | *Anthonomus nigrinus* |  |  |  |  |  |  |  |  | 1 |  |  |  |
|  |  | | *Ellescus ephippiatus* |  |  |  |  |  |  | 1 |  |  |  |  |  |
|  |  | | *Isochnus arcticus* |  | 24 | 1 |  |  |  |  |  |  |  |  |  |
|  | Cyclominae | |  |  |  |  |  |  |  |  |  |  |  |  |  |
|  |  | | *Listronotus humilis* |  |  |  |  |  |  | 1* |  |  |  | 1* |  |
|  | Dryophthorinae | |  |  |  |  |  |  |  |  |  |  |  |  |  |
|  |  | | *Sphenophorus costipennis* |  |  |  |  |  |  |  |  |  |  |  | 1 |
|  | Entiminae | |  |  |  |  |  |  |  |  |  |  |  |  |  |
|  |  | | *Lepidophorus lineatocollis* |  |  |  |  |  | 1 |  |  | 27 |  |  |  |
|  |  | | *Otiorhynchus ovatus* |  |  |  |  |  |  |  |  |  |  | 2 |  |
|  |  | | *Sciaphilus asperatus* |  |  |  |  |  |  |  |  |  |  |  | 1 |
|  |  | | *Sitona lineellus* |  |  |  |  |  |  |  |  | 1 |  |  |  |
|  | Erirhininae | |  |  |  |  |  |  |  |  |  |  |  |  |  |
|  |  | | *Grypus equiseti* |  |  |  |  |  |  |  |  |  |  |  | 32 |
|  |  | | *Notaris aethiops* |  |  |  |  |  | 1 | 2 |  | 3 | 6 |  |  |
|  |  | | *Procas lecontei* |  |  |  |  |  |  |  |  |  | 1 |  |  |
|  | Molytinae | |  |  |  |  |  |  |  |  |  |  |  |  |  |
|  |  | | *Hylobius congener* |  |  |  |  |  |  |  |  |  |  | 63 |  |
|  |  | | *Hypera diversipunctata* |  |  |  |  | 1* | 1 |  |  |  |  |  |  |
|  |  | | *Hypera* sp.*C* |  |  | 1 |  |  |  |  |  |  |  |  |  |
|  |  | | *Hypera* sp.*T* |  |  |  |  |  | 1 |  |  |  |  |  |  |
|  |  | | *Lepyrus gemellus* |  |  |  |  | 1 |  |  |  |  |  |  |  |
|  |  | | *Lepyrus nordenskioeldi* |  |  |  |  | 8 |  |  |  |  |  |  |  |
|  |  | | *Lepyrus nordenskioeldi* |  |  | 2 |  |  |  |  |  |  |  |  |  |
|  |  | | *Lepyrus* sp.*C* |  |  | 10 |  |  |  |  |  |  |  |  |  |
|  |  | | *Lepyrus* sp.*H* |  |  |  |  |  |  | 21 |  |  |  |  |  |
|  |  | | *Lepyrus* sp.*N* |  |  |  |  |  |  |  |  | 4 |  |  |  |
|  |  | | *Lepyrus* sp.*T* |  |  |  |  |  | 1 |  |  |  |  |  |  |
|  |  | | *Pissodes nemorensis* |  |  |  |  |  |  |  |  |  |  | 5* |  |
|  | Scolytinae | |  |  |  |  |  |  |  |  |  |  |  |  |  |
|  |  | | *Pityokteines* sp.1 |  |  |  |  |  |  |  |  |  | 1 |  |  |
|  |  | | *Scolytine* sp. |  |  |  |  |  |  |  |  |  |  | 1 |  |
|  |  | | *Scolytine* sp. 2 |  |  |  |  |  |  |  |  |  | 1 |  |  |
| Elateridae | | |  |  |  |  |  |  |  |  |  |  |  |  |  |
|  | Elaterinae | |  |  |  |  |  |  |  |  |  |  |  |  |  |
|  |  | | *Agriotes limosus* |  |  |  |  |  |  |  |  | 2 |  | 5 |  |
|  |  | | *Ampedus nigrinus* |  |  |  |  |  |  |  |  | 5 |  |  |  |
|  |  | | *Ampedus pullus* |  |  |  |  |  |  |  |  | 9 |  |  |  |
|  |  | | *Dalopius pallidus* |  |  |  |  |  |  |  |  |  |  |  | 21 |
|  |  | | *Sericus incongruus* |  |  |  |  |  |  |  |  | 2 | 5 | 4 |  |
|  | Negastriinae | |  |  |  |  |  |  |  |  |  |  |  |  |  |
|  |  | | *Negastrius arnetti* |  |  |  |  |  |  |  |  |  |  |  | 1 |
|  |  | | *Neohypdonus gentilis* |  |  |  |  |  |  |  |  |  |  |  | 4* |
|  | Prosterinae | |  |  |  |  |  |  |  |  |  |  |  |  |  |
|  |  | | *Ascoliocerus sanborni* |  |  |  |  |  | 16 |  |  |  |  |  |  |
|  |  | | *Beckerus appressus* |  |  |  |  |  |  |  | 3 |  |  |  |  |
|  |  | | *Eanus decoratus* |  |  |  |  |  | 2 | 5 | 1 |  |  | 1 |  |
|  |  | | *Eanus maculipennis* |  |  |  |  |  |  |  | 1 |  |  | 3 |  |
|  |  | | *Hypnoidus abbreviatus* |  |  |  |  |  |  |  |  |  |  |  | 5 |
|  |  | | *Hypnoidus bicolor* |  |  |  |  |  | 4 |  | 28 | 1 | 5 | 1 | 1 |
|  |  | | *Hypnoidus rivularius* |  |  |  |  |  | 1 |  |  | 1 |  |  |  |
|  |  | | *Limonius aeger* |  |  |  |  |  |  |  |  |  |  | 3 |  |
|  |  | | *Pseudanostirus ochreipennis* |  |  |  |  |  |  |  |  |  | 2 |  |  |
|  |  | | *Pseudanostirus triundulatus* |  |  |  |  |  |  |  |  |  | 1 | 5 | 1 |
|  |  | | *Selatosomus aeripennis* |  |  |  |  |  |  |  |  |  | 15 |  |  |
|  |  | | *Setasomus aratus* |  |  |  |  |  |  |  |  |  | 1 |  |  |
|  |  | | *Sylvanelater mendax* |  |  |  |  |  |  |  |  | 1 |  |  |  |
| Eucinetidae | | |  |  |  |  |  |  |  |  |  |  |  |  |  |
|  |  | | *Eucinetus haemorrhoidalis* |  |  |  |  |  |  |  |  | 6 |  |  |  |
| Histeridae | | |  |  |  |  |  |  |  |  |  |  |  |  |  |
|  |  | | *Histeridae* sp. |  |  |  |  |  |  |  |  |  |  | 1 |  |
| Hydraenidae | | |  |  |  |  |  |  |  |  |  |  |  |  |  |
|  |  | | *Hydraena* sp. |  |  |  |  |  |  | 1 |  |  |  |  |  |
| Hydrophilidae | | |  |  |  |  |  |  |  |  |  |  |  |  |  |
|  | Sphaeridiinae | |  |  |  |  |  |  |  |  |  |  |  |  |  |
|  |  | | *Cercyon* sp. |  |  |  |  |  |  |  |  | 1 |  |  |  |
|  |  | | *Cercyon* sp. 2 |  |  |  |  |  |  |  |  |  |  | 73 |  |
|  |  | | *Cercyon* sp. 3 |  |  |  |  |  |  |  |  |  |  |  | 1 |
|  |  | | *Megasternum* sp. |  |  |  |  |  |  |  |  |  |  |  | 1 |
|  |  | | *Phaenonotum* sp. |  |  |  |  |  |  |  |  | 1 |  |  |  |
|  |  | | *Phaenonotum* sp.2 |  |  |  |  |  |  |  | 1 |  |  |  |  |
| Lampyridae | | |  |  |  |  |  |  |  |  | 1 |  |  |  | 4 |
|  |  | | *Lampyrid* sp. |  |  |  |  |  |  |  |  |  |  |  | 1 |
|  |  | | *Lampyrid* sp. 2 |  |  |  |  |  |  |  | 1 |  |  |  |  |
|  |  | | *Lampyrid* sp. 3 |  |  |  |  |  |  |  |  |  |  |  | 3 |
| Latridiidae | | |  |  |  |  |  |  |  |  |  |  |  |  |  |
|  | Corticariinae | |  |  |  |  |  |  |  |  |  |  |  |  |  |
|  |  | | *Corticaria* sp. |  |  |  |  |  |  |  |  |  |  | 4 |  |
|  |  | | *Corticaria* sp. 2 |  |  |  |  | 1 |  |  |  |  |  |  |  |
|  |  | | *Corticaria* sp.3 |  |  |  |  |  | 20 |  |  |  |  |  |  |
|  |  | | *Corticaria* sp. 4 |  |  |  |  |  | 2 |  |  |  |  |  |  |
|  |  | | *Corticaria* sp. 4 |  |  |  |  |  | 2 |  |  |  |  |  |  |
|  |  | | *Corticaria* sp. 5 |  |  |  |  |  |  |  |  | 18 |  |  |  |
|  |  | | *Corticarina* sp. |  |  |  |  |  |  |  |  | 8 |  |  |  |
|  |  | | *Melanophthalma* sp. |  |  |  |  |  |  |  |  | 9 |  |  |  |
|  |  | | *Melanophthalma* sp. 2 |  |  |  |  |  |  |  |  |  | 26 |  |  |
|  |  | | *Melanophthalma* sp. 3 |  |  |  |  |  |  |  |  |  |  | 1 |  |
|  |  | | *Melanophthalma* sp. 4 |  |  |  |  |  |  |  |  |  |  |  | 7 |
|  | Latridiinae | |  |  |  |  |  |  |  |  |  |  |  |  |  |
|  |  | | *Enicmus* sp. |  | 21 |  |  |  |  |  |  |  |  |  |  |
|  |  | | *Enicmus* sp. 2 |  |  |  |  |  |  |  |  |  |  | 7 |  |
|  |  | | *Latridius* sp. 3 |  |  | 3 |  |  |  |  |  |  |  |  |  |
|  |  | | *Latridius* sp. 4 |  |  |  |  |  |  |  |  | 1 |  |  |  |
| Leiodidae | | |  |  |  |  |  |  |  |  |  |  |  |  |  |
|  | Coloninae | |  |  |  |  |  |  |  |  |  |  |  |  |  |
|  |  | | *Catops* sp. |  |  |  |  |  |  |  | 1 |  |  |  |  |
|  |  | | *Colon asperatum* |  |  |  |  |  |  |  |  |  |  |  | 26 |
|  |  | | *Colon bidentatum* |  |  |  |  |  |  |  |  |  |  |  | 1 |
|  |  | | *Colon dentatum* |  |  |  |  |  |  |  |  |  |  |  | 4 |
|  |  | | *Colon magnicolle* |  |  |  |  |  |  |  |  |  |  |  | 1 |
|  |  | | *Colon oblongum* |  |  |  |  |  |  |  |  | 7* | 27* |  | 26 |
|  |  | | *Colon politum* |  |  |  |  |  |  |  |  |  |  |  | 7* |
|  | Leiodinae | |  |  |  |  |  |  |  |  |  |  |  |  |  |
|  |  | | *Agathidium* sp. |  |  |  |  |  |  |  |  | 1 |  |  |  |
|  |  | | *Agathidium* sp. 2 |  |  |  |  |  |  | 2 |  |  |  |  |  |
|  |  | | *Agathidium* sp. 3 |  |  |  |  |  |  |  |  | 1 |  |  |  |
|  |  | | *Agathidium* sp. 4 |  |  |  |  |  | 6 |  |  |  |  |  |  |
|  |  | | *Agathidium* sp. 5 |  |  |  |  |  | 2 |  |  |  |  |  |  |
|  |  | | *Agathidium* sp. 6 |  |  |  |  |  |  |  |  | 5 |  |  |  |
|  |  | | *Anisotoma* sp. 7 |  |  |  |  |  |  |  |  | 2 |  |  |  |
|  |  | | *Hydnobius* sp. |  |  |  |  |  |  |  |  |  |  |  | 12 |
|  |  | | *Leiodes assimilis* |  |  |  |  |  |  |  |  |  |  |  | 7 |
|  |  | | *Leiodes neglecta* |  |  |  |  |  |  |  |  |  |  |  | 23 |
|  |  | | *Leiodes punctostriata* |  |  |  |  |  |  |  |  | 2 | 24 |  | 2 |
|  |  | | *Leiodes* sp. |  |  |  |  |  |  |  |  |  |  |  | 2 |
|  |  | | *Leiodes* sp. 2 |  |  |  |  |  |  |  |  |  |  |  | 32 |
|  |  | | *Leiodes* sp. 3 |  |  |  |  |  |  |  |  | 1 |  |  |  |
| Lucanidae | | |  |  |  |  |  |  |  |  |  |  |  |  |  |
|  | Lucaninae | |  |  |  |  |  |  |  |  |  |  |  |  |  |
|  |  | | *Platycerus* sp. |  |  |  |  |  |  |  |  |  | 1 |  |  |
| Mordellidae | | |  |  |  |  |  |  |  |  |  |  |  |  |  |
|  |  | | *Mordellochroa scapularis* |  |  |  |  |  |  |  |  | 1* |  |  |  |
| Nitidulidae | | |  |  |  |  |  |  |  |  |  |  |  |  |  |
|  | Carpophilinae | |  |  |  |  |  |  |  |  |  |  |  |  |  |
|  |  | | *Epuraea* sp. |  |  |  |  |  |  |  |  |  |  | 1 |  |
|  |  | | *Epuraea* sp. 2 |  |  |  |  |  |  | 10 |  |  |  |  |  |
|  |  | | *Epuraea* sp. 3 |  |  |  |  |  |  |  |  |  |  |  | 1 |
|  |  | | *Epuraea* sp. 4 |  |  |  |  |  |  |  |  | 35 |  |  |  |
|  |  | | *Epuraea* sp. 5 |  |  |  |  |  |  |  | 2 |  |  |  |  |
|  |  | | *Epuraea* sp. 6 |  |  |  |  |  | 2 |  |  |  |  |  |  |
|  |  | | *Epuraea* sp. 7 |  |  |  |  |  |  |  |  |  | 83 |  |  |
|  | Cryptarchinae | |  |  |  |  |  |  |  |  |  |  |  |  |  |
|  |  | | *Glischrochilus siepmanni* |  |  |  |  |  |  |  |  |  | 2* |  |  |
| Phalacridae | | |  |  |  |  |  |  |  |  |  |  |  |  |  |
|  |  | | *Stilbus* sp. |  |  |  |  |  |  |  |  |  |  |  | 2 |
| Ptiliidae | | |  |  |  |  |  |  |  |  |  |  |  |  |  |
|  |  | | *Ptiliid* sp. |  |  |  |  |  |  |  |  |  |  |  | 9 |
|  |  | | *Ptiliid* sp. 2 |  |  |  |  |  |  |  |  |  | 1 |  |  |
| Ptinidae | | |  |  |  |  |  |  |  |  |  |  |  |  |  |
|  | Dorcatominae | |  |  |  |  |  |  |  |  |  |  |  |  |  |
|  |  | | *Caenocara* sp. |  |  |  |  |  |  | 5 |  |  |  |  |  |
|  |  | | *Caenocara* sp. 2 |  |  |  |  |  | 1 |  |  |  |  |  |  |
|  |  | | *Caenocara* sp. 3 |  |  |  |  |  |  |  |  |  | 3 |  |  |
| Pythidae | | |  |  |  |  |  |  |  |  |  |  |  | 1 |  |
|  |  | | *Priognathus monilicornis* |  |  |  |  |  |  |  |  |  |  | 1* |  |
| Scarabaeidae | | |  |  |  |  |  |  |  |  |  |  |  |  |  |
|  | Aphodiinae | |  |  |  |  |  |  |  |  |  |  |  |  |  |
|  |  | | *Aegialia lacustris* |  |  |  |  |  |  | 1 |  |  |  |  | 1 |
| Scirtidae | | |  |  |  |  |  |  |  |  |  |  |  |  |  |
|  |  | | *Cyphon* sp. |  |  |  |  |  |  |  |  |  |  | 1 |  |
|  |  | | *Cyphon* sp. 2 |  |  |  |  |  |  |  |  |  |  |  | 3 |
|  |  | | *Cyphon* sp. 3 |  |  |  |  |  |  |  |  | 7 |  |  |  |
| Scydmaenidae | | |  |  |  |  |  |  |  |  |  |  |  |  |  |
|  | Scydmaeninae | |  |  |  |  |  |  |  |  |  |  |  |  |  |
|  |  | | *Scydmaenid* sp. |  |  |  |  |  |  |  |  |  | 1 |  |  |
| Silphidae | | |  |  |  |  |  |  |  |  |  |  |  |  |  |
|  |  | | *Nicrophorus defodiens* |  |  |  |  |  |  |  |  | 2 | 1 |  |  |
|  |  | | *Thanatophilus lapponicus* |  |  |  |  |  | 4 |  |  |  |  |  |  |
|  |  | | *Thanatophilus sagax* |  |  |  |  |  | 19 |  |  | 64 | 2 |  |  |
| Staphylinidae | | |  |  |  |  |  |  |  |  |  |  |  |  |  |
|  | Aleocharinae | |  |  |  |  |  |  |  |  |  |  |  |  |  |
|  |  | | *Aleochara assiniboin* |  |  |  |  |  |  |  |  |  | 1* |  |  |
|  |  | | *Aleocharine* sp. 1 |  | 2 |  |  |  |  |  |  |  |  |  |  |
|  |  | | *Aleocharine* sp. 2 |  |  |  |  |  |  |  |  |  |  | 16 |  |
|  |  | | *Aleocharine* sp. 3 |  |  |  |  |  |  |  |  |  |  | 2 |  |
|  |  | | *Aleocharine* sp. 4 |  |  |  |  |  |  |  |  |  | 3 |  |  |
|  |  | | *Aleocharine* sp. 5 |  |  |  |  |  |  |  |  |  |  |  | 8 |
|  |  | | *Aleocharine* sp. 6 |  |  |  |  |  |  |  |  | 3 |  |  |  |
|  |  | | *Aleocharine* sp. 7 |  |  |  |  |  |  |  |  | 1 |  |  |  |
|  |  | | *Aleocharine* sp. 8 |  |  |  |  |  |  | 2 |  |  |  |  |  |
|  |  | | *Aleocharine* sp. 9 |  |  |  |  | 1 |  |  |  |  |  |  |  |
|  |  | | *Aleocharine* sp. 10 |  |  |  |  |  |  |  |  |  |  |  | 4 |
|  |  | | *Aleocharine* sp. 11 |  |  |  |  |  |  |  |  | 29 |  |  |  |
|  |  | | *Aleocharine* sp. 12 |  |  |  |  |  |  |  | 4 |  |  |  |  |
|  |  | | *Aleocharine* sp. 13 |  |  |  |  |  | 1 |  |  |  |  |  |  |
|  |  | | *Aleocharine* sp. 14 |  |  |  |  |  |  |  |  |  | 34 |  |  |
|  |  | | *Aleocharine* sp. 15 |  |  |  |  |  |  | 1 |  |  |  |  |  |
|  |  | | *Aleocharine* sp. 16 |  |  |  | 1 |  |  |  |  |  |  |  |  |
|  |  | | *Aleocharine* sp. 17 |  |  |  |  | 1 |  |  |  |  |  |  |  |
|  |  | | *Aleocharine* sp. 18 |  |  |  |  |  |  |  |  |  |  |  | 20 |
|  |  | | *Aleocharine* sp. 19 |  |  |  |  |  |  |  |  | 4 |  |  |  |
|  |  | | *Aleocharine* sp. 20 |  |  |  |  |  | 1 |  |  |  |  |  |  |
|  |  | | *Aleocharine* sp. 21 |  |  |  |  |  |  |  |  |  | 4 |  |  |
|  |  | | *Aleocharine* sp. 22 |  |  |  |  |  |  | 2 |  |  |  |  |  |
|  |  | | *Aleocharine* sp. 23 |  |  |  |  | 1 |  |  |  |  |  |  |  |
|  |  | | *Aleocharine* sp. 24 |  |  |  |  |  |  |  |  |  |  |  | 1 |
|  |  | | *Aleocharine* sp. 25 |  |  |  |  |  |  |  |  | 5 |  |  |  |
|  |  | | *Aleocharine* sp. 26 |  |  |  |  |  |  |  | 5 |  |  |  |  |
|  |  | | *Aleocharine* sp. 27 |  |  |  |  |  | 1 |  |  |  |  |  |  |
|  |  | | *Aleocharine* sp. 28 |  |  |  |  |  |  |  |  |  | 6 |  |  |
|  |  | | *Aleocharine* sp. 29 |  |  |  |  | 1 |  |  |  |  |  |  |  |
|  |  | | *Aleocharine* sp. 30 |  |  |  |  |  |  |  |  |  |  |  | 1 |
|  |  | | *Aleocharine* sp. 31 |  |  |  |  |  |  |  |  | 12 |  |  |  |
|  |  | | *Aleocharine* sp. 32 |  |  |  |  |  |  |  | 1 |  |  |  |  |
|  |  | | *Aleocharine* sp. 33 |  |  |  |  |  | 4 |  |  |  |  |  |  |
|  |  | | *Aleocharine* sp. 34 |  |  |  |  |  |  |  |  |  | 1 |  |  |
|  |  | | *Aleocharine* sp. 35 |  |  |  |  | 1 |  |  |  |  |  |  |  |
|  |  | | *Aleocharine* sp. 36 |  |  |  |  |  |  |  |  |  |  |  | 5 |
|  |  | | *Aleocharine* sp. 37 |  |  |  |  |  |  |  |  | 4 |  |  |  |
|  |  | | *Aleocharine* sp. 38 |  |  |  |  |  |  |  | 3 |  |  |  |  |
|  |  | | *Aleocharine* sp. 39 |  |  |  |  |  |  |  |  |  | 9 |  |  |
|  |  | | *Aleocharine* sp. 40 |  |  |  |  | 1 |  |  |  |  |  |  |  |
|  |  | | *Aleocharine* sp. 41 |  |  |  |  |  |  |  |  |  |  |  | 3 |
|  |  | | *Aleocharine* sp. 42 |  |  |  |  |  |  |  |  | 1 |  |  |  |
|  |  | | *Aleocharine* sp. 43 |  |  |  |  |  |  |  | 29 |  |  |  |  |
|  |  | | *Aleocharine* sp. 44 |  |  |  |  |  |  |  |  |  | 2 |  |  |
|  |  | | *Aleocharine* sp. 45 |  |  |  |  |  |  |  |  |  |  |  | 4 |
|  |  | | *Aleocharine* sp. 46 |  |  |  |  |  |  |  | 1 |  |  |  |  |
|  |  | | *Aleocharine* sp. 47 |  |  |  |  |  |  |  |  |  |  |  | 7 |
|  |  | | *Aleocharine* sp. 48 |  |  |  |  |  |  |  |  | 19 |  |  |  |
|  |  | | *Aleocharine* sp. 49 |  |  |  |  |  |  |  |  |  | 7 |  |  |
|  |  | | *Aleocharine* sp. 50 |  |  |  |  |  |  |  |  |  |  |  | 1 |
|  |  | | *Aleocharine* sp. 51 |  |  |  |  |  |  |  |  | 4 |  |  |  |
|  |  | | *Aleocharine* sp. 52 |  |  |  |  |  |  |  |  |  | 7 |  |  |
|  |  | | *Gnypeta ashei* | 14 |  |  |  |  |  |  |  |  |  |  |  |
|  |  | | *Liogluta nigropolita* |  |  |  | 1 |  |  |  |  |  |  |  |  |
|  | Euaesthetinae | |  |  |  |  |  |  |  |  |  |  |  |  |  |
|  |  | | *Euaesthetus* sp. |  |  |  |  |  |  |  |  |  |  |  | 13 |
|  | Omaliinae | |  |  |  |  |  |  |  |  |  |  |  |  |  |
|  |  | | *Acidota quadrata* |  |  | 12 |  |  |  |  |  |  |  |  |  |
|  |  | | *Eusphalerum* sp. |  |  |  |  |  |  |  |  |  | 1 |  |  |
|  |  | | *Eusphalerum* sp. 2 |  |  |  |  |  | 34 |  |  |  |  |  |  |
|  |  | | *Olophrum latum* |  |  | 7 |  |  |  |  |  |  |  |  |  |
|  |  | | *Omaliine* sp. 1 |  |  |  |  |  | 1 |  |  |  |  |  |  |
|  |  | | *Omaliine* sp. 2 |  |  |  |  |  |  |  |  |  |  | 1 |  |
|  |  | | *Omaliine* sp. 3 |  |  |  |  |  |  | 5 |  |  |  |  |  |
|  |  | | *Omaliine* sp. 4 |  |  |  |  | 3 |  |  |  |  |  |  |  |
|  |  | | *Omaliine* sp. 5 |  |  |  |  |  |  |  |  |  |  |  | 3 |
|  |  | | *Omaliine* sp. 6 |  |  |  |  |  |  |  |  | 1 |  |  |  |
|  |  | | *Omaliine* sp. 7 |  |  |  |  |  |  |  | 10 |  |  |  |  |
|  |  | | *Omaliine* sp. 8 |  |  |  |  |  | 1 |  |  |  |  |  |  |
|  |  | | *Omaliine* sp. 9 |  |  |  |  |  |  |  |  |  | 3 |  |  |
|  |  | | *Omaliine* sp. 10 |  |  |  |  |  |  | 1 |  |  |  |  |  |
|  |  | | *Omaliine* sp. 11 |  |  |  |  |  |  |  |  |  |  |  | 2 |
|  |  | | *Omaliine* sp. 12 |  |  |  |  |  |  |  |  | 1 |  |  |  |
|  |  | | *Omaliine* sp. 13 |  |  |  |  |  |  |  | 3 |  |  |  |  |
|  |  | | *Omaliine* sp. 14 |  |  |  |  |  | 1 |  |  |  |  |  |  |
|  |  | | *Omaliine* sp. 15 |  |  |  |  |  |  |  |  |  | 1 |  |  |
|  |  | | *Omaliine* sp. 16 |  |  |  |  |  |  | 3 |  |  |  |  |  |
|  |  | | *Omaliine* sp. 17 |  |  |  |  |  |  |  |  | 3 |  |  |  |
|  |  | | *Omaliine* sp. 18 |  |  |  |  |  |  |  | 1 |  |  |  |  |
|  |  | | *Omaliine* sp. 19 |  |  |  |  |  | 1 |  |  |  |  |  |  |
|  |  | | *Omaliine* sp. 20 |  |  |  |  |  |  |  | 2 |  |  |  |  |
|  |  | | *Omaliine* sp. 21 |  |  |  |  |  | 3 |  |  |  |  |  |  |
|  |  | | *Omaliine* sp. 22 |  |  |  |  |  | 3 |  |  |  |  |  |  |
|  |  | | *Omaliine* sp. 23 |  |  |  |  |  | 1 |  |  |  |  |  |  |
|  |  | | *Omaliine* sp. 24 |  |  |  |  |  |  |  |  |  | 2 |  |  |
|  |  | | *Omaliine* sp. 25 |  |  |  |  |  |  |  |  |  | 1 |  |  |
|  | Oxyporinae | |  |  |  |  |  |  |  |  |  |  |  |  |  |
|  |  | | *Oxyporus* sp. |  |  |  |  |  |  |  |  |  | 1 |  |  |
|  |  | | *Oxyporus* sp. 2 |  |  |  |  |  |  |  |  |  |  |  | 8 |
|  | Paederinae | |  |  |  |  |  |  |  |  |  |  |  |  |  |
|  |  | | *Paederine* sp. |  |  |  |  |  |  |  |  | 34 |  |  |  |
|  |  | | *Paederine* sp. 2 |  |  |  |  |  |  |  |  |  | 42 |  |  |
|  | Piestinae | |  |  |  |  |  |  |  |  |  |  |  |  |  |
|  |  | | *Piestine* sp. |  |  |  |  |  |  |  |  |  |  | 1 |  |
|  | Proteininae | |  |  |  |  |  |  |  |  |  |  |  |  |  |
|  |  | | *Proteinine* sp. |  |  |  |  |  |  |  |  | 1 |  |  |  |
|  |  | | *Proteinus* sp. 2 |  |  |  |  |  |  |  | 3 |  |  |  |  |
|  | Pselaphinae | |  |  |  |  |  |  |  |  |  |  |  |  |  |
|  |  | | *Pselaphine* sp. |  |  |  |  |  |  |  |  |  |  | 1 |  |
|  |  | | *Pselaphine* sp. 2 |  |  |  |  |  |  |  |  |  |  |  | 1 |
|  |  | | *Pselaphine* sp. 3 |  |  |  |  |  |  |  |  | 2 |  |  |  |
|  |  | | *Reichenbachia* sp. |  |  |  |  |  |  |  |  |  |  |  | 1 |
|  | Scaphidiinae | |  |  |  |  |  |  |  |  |  |  |  |  |  |
|  |  | | *Baeocera* sp. |  |  |  |  |  |  |  |  |  |  | 6 |  |
|  | Scydmaeninae | |  |  |  |  |  |  |  |  |  |  |  |  |  |
|  |  | | *Brachycepsis* sp. |  |  |  |  |  |  | 2 |  |  |  |  |  |
|  |  | | *Scydmaenid* sp. |  |  |  |  |  |  |  | 6 |  |  |  |  |
|  | Staphylininae | |  |  |  |  |  |  |  |  |  |  |  |  |  |
|  |  | | *Quedius fellmanii* |  |  |  |  | 2 |  |  |  |  |  |  |  |
|  |  | | *Staphylinine* sp. |  |  |  |  |  |  |  |  |  |  |  | 1 |
|  |  | | *Staphylinine* sp. 2 |  |  |  |  |  |  | 4 |  |  |  |  |  |
|  |  | | *Staphylinine* sp. 3 |  |  |  |  |  |  |  |  | 8 |  |  |  |
|  |  | | *Staphylinine* sp. 4 |  |  |  |  |  | 1 |  |  |  |  |  |  |
|  |  | | *Staphylinine* sp. 5 |  |  |  |  |  |  |  |  |  | 5 |  |  |
|  |  | | *Staphylinine* sp. 6 |  |  |  |  |  |  | 2 |  |  |  |  |  |
|  |  | | *Staphylinine* sp. 7 |  |  |  |  |  |  |  |  | 4 |  |  |  |
|  |  | | *Staphylinine* sp. 8 |  |  |  |  |  | 1 |  |  |  |  |  |  |
|  |  | | *Staphylinine* sp. 9 |  |  |  |  |  |  |  |  |  | 9 |  |  |
|  |  | | *Staphylinine* sp. 10 |  |  |  |  |  |  |  |  |  |  |  | 2 |
|  |  | | *Staphylinine* sp. 11 |  |  |  |  |  |  |  |  | 9 |  |  |  |
|  |  | | *Staphylinine* sp. 12 |  |  |  |  |  | 4 |  |  |  |  |  |  |
|  |  | | *Staphylinine* sp. 13 |  |  |  |  |  |  |  |  |  | 1 |  |  |
|  |  | | *Staphylinine* sp. 14 |  |  |  |  |  |  |  |  |  |  |  | 4 |
|  |  | | *Staphylinine* sp. 15 |  |  |  |  |  |  |  |  | 4 |  |  |  |
|  |  | | *Staphylinine* sp. 16 |  |  |  |  |  | 6 |  |  |  |  |  |  |
|  |  | | *Staphylinine* sp. 17 |  |  |  |  |  |  |  |  |  |  |  | 2 |
|  |  | | *Staphylinine* sp. 18 |  |  |  |  |  | 7 |  |  |  |  |  |  |
|  |  | | *Staphylinine* sp. 19 |  |  |  |  |  |  |  |  |  |  |  | 7 |
|  |  | | *Staphylinine* sp. 20 |  |  |  |  |  |  |  |  |  |  |  | 2 |
|  |  | | *Staphylinine* sp. 21 |  |  |  |  |  |  |  |  |  |  |  | 5 |
|  |  | | *Staphylinine* sp. 22 |  |  |  |  |  |  |  |  |  |  |  | 1 |
|  |  | | *Tympanophorus puncticollis* |  |  |  |  |  |  |  |  |  |  |  | 1 |
|  | Steninae | |  |  |  |  |  |  |  |  |  |  |  |  |  |
|  |  | | *Stenus* sp. |  | 1 |  |  |  |  |  |  |  |  |  |  |
|  |  | | *Stenus* sp. 2 |  |  | 3 |  |  |  |  |  |  |  |  |  |
|  |  | | *Stenus* sp. 3 |  |  |  |  |  |  |  |  |  |  | 2 |  |
|  |  | | *Stenus* sp. 4 |  |  |  |  |  |  | 1 |  |  |  |  |  |
|  |  | | *Stenus* sp. 5 |  |  |  |  | 4 |  |  |  |  |  |  |  |
|  |  | | *Stenus* sp. 6 |  |  |  |  |  |  |  |  |  |  |  | 16 |
|  |  | | *Stenus* sp. 7 |  |  |  |  |  |  |  |  | 21 |  |  |  |
|  |  | | *Stenus* sp. 8 |  |  |  |  |  |  |  | 2 |  |  |  |  |
|  |  | | *Stenus* sp. 9 |  |  |  |  |  | 1 |  |  |  |  |  |  |
|  |  | | *Stenus* sp. 10 |  |  |  |  |  |  |  |  |  | 6 |  |  |
|  |  | | *Stenus frigidus* |  |  | 1 |  |  |  |  |  |  |  |  |  |
|  | Tachyporinae | |  |  |  |  |  |  |  |  |  |  |  |  |  |
|  |  | | *Bryophacis arcticus* |  |  |  |  | 7 |  | 11 |  |  |  |  |  |
|  |  | | *Bryophacis smetanai* |  |  |  |  |  |  |  |  |  | 1 |  |  |
|  |  | | *Ischnosoma longicorne* |  |  |  |  |  |  |  |  |  | 3 |  |  |
|  |  | | *Ischnosoma pictum* |  |  |  |  |  |  | 1 |  |  |  | 10 |  |
|  |  | | *Ischnosoma splendidum* |  |  |  |  |  |  |  |  |  | 3 |  |  |
|  |  | | *Lordithon fungicola* |  |  |  |  |  |  |  |  |  | 3 |  |  |
|  |  | | *Mycetophorus nigrans* |  |  |  |  | 3* |  | 5 |  |  | 1 |  |  |
|  |  | | *Mycetoporus smetanai* |  |  |  |  |  |  |  |  |  | 1 |  |  |
|  |  | | *Tachinus basalis* |  |  |  |  |  | 1 |  |  |  |  |  |  |
|  |  | | *Tachinus elongatus* |  |  |  |  |  | 3 | 3 |  |  | 6 |  |  |
|  |  | | *Tachyporus borealis* |  |  |  |  |  |  |  |  |  | 7 |  |  |
|  |  | | *Tachyporus nimbicola* |  |  |  |  |  |  | 1* |  |  |  |  |  |
|  |  | | *Tachyporus nitidulus* |  |  |  |  |  |  |  |  |  | 3 |  |  |
|  |  | | *Tachyporus rulomus* |  |  |  |  |  |  |  |  |  | 5 |  |  |
|  |  | | *Tachyporine* sp. |  |  |  | 1 |  |  |  |  |  |  |  |  |
|  |  | | *Tachyporine* sp. 2 |  |  |  |  |  |  |  |  |  |  |  | 4 |
|  |  | | *Tachyporine* sp. 3 |  |  |  |  |  |  |  |  | 3 |  |  |  |
|  |  | | *Tachyporine* sp. 4 |  |  |  |  |  |  |  | 23 |  |  |  |  |
|  |  | | *Tachyporine* sp. 5 |  |  |  |  |  |  |  |  |  |  |  | 6 |
|  |  | | *Tachyporine* sp. 6 |  |  |  |  |  |  |  |  | 7 |  |  |  |
|  |  | | *Tachyporine* sp. 7 |  |  |  |  |  |  |  | 1 |  |  |  |  |
|  |  | | *Tachyporine* sp. 8 |  |  |  |  |  |  |  |  |  | 1 |  |  |
|  |  | | *Tachyporine* sp. 9 |  |  |  |  |  |  |  |  |  |  |  | 9 |
|  |  | | *Tachyporine* sp. 10 |  |  |  |  |  |  |  |  | 8 |  |  |  |
|  |  | | *Tachyporine* sp. 11 |  |  |  |  |  |  |  | 1 |  |  |  |  |
|  |  | | *Tachyporine* sp. 12 |  |  |  |  |  |  | 1 |  |  |  |  |  |
|  |  | | *Tachyporine* sp. 13 |  |  |  |  |  |  |  |  |  |  |  | 1 |
|  |  | | *Tachyporine* sp. 14 |  |  |  |  |  |  |  |  | 3 |  |  |  |
|  |  | | *Tachyporine* sp. 15 |  |  |  |  |  |  |  | 3 |  |  |  |  |
|  |  | | *Tachyporine* sp. 16 |  |  |  |  |  |  |  |  |  |  |  | 3 |
|  |  | | *Tachyporine* sp. 17 |  |  |  |  |  |  |  |  | 12 |  |  |  |
|  |  | | *Tachyporine* sp. 18 |  |  |  |  |  |  |  | 10 |  |  |  |  |
|  |  | | *Tachyporine* sp. 19 |  |  |  |  |  |  |  |  |  | 1 |  |  |
|  |  | | *Tachyporine* sp. 20 |  |  |  |  |  |  |  |  | 1 |  |  |  |
|  |  | | *Tachyporine* sp. 21 |  |  |  |  |  |  |  |  |  | 1 |  |  |
|  |  | | *Tachyporine* sp. 22 |  |  |  |  |  |  |  | 1 |  |  |  |  |
|  |  | | *Tachyporine* sp. 23 |  |  |  |  |  |  |  |  |  | 10 |  |  |
| Tenebrionidae | | |  |  |  |  |  |  |  |  |  |  |  |  |  |
|  | Stenochiinae | |  |  |  |  |  |  |  |  |  |  |  |  |  |
|  |  | | *Upis ceramboides* |  |  |  |  |  |  |  |  |  | 2 |  |  |
| Grand Total | | |  | 14 | 702 | 1696 | 420 | 1180 | 701 | 674 | 423 | 998 | 714 | 381 | 1159 |
