# Supplementary material for: Drivers and Patterns of Ground-Dwelling Beetle Biodiversity across Northern Canada
Source: PLoS One. 2015 Apr 22;10(4):e0122163. doi: 10.1371/journal.pone.0122163 (PMC4406721; doi:10.1371/journal.pone.0122163)
Supplement: S2 Table — Molluscivores, Collembola and Mite Specialist Predators, Generalist Omnivores, Non-feeding Adults, Xylophages, and Micropolyvores have been omitted for clarity because their contributions to the biomass are extremely small (< 1% of total) in all locations. (DOCX) [file pone.0122163.s002.docx]

| **Functional Group** | **HAZ** | **BAN** | **CAM** | **IQA** | **KUG** | **TOM** | **CHU** | **SCH** | **NOR** | **YEL** | **GOB** | **MOO** |
| --- | --- | --- | --- | --- | --- | --- | --- | --- | --- | --- | --- | --- |
| Generalist Predator | 1.00 | 0.31 | 0.36 | 0.91 | 0.76 | 0.88 | 0.88 | 0.79 | 0.72 | 0.76 | 0.60 | 0.94 |
| **Carnivores (All)** | **1.00** | **0.31** | **0.36** | **0.91** | **0.77** | **0.88** | **0.88** | **0.79** | **0.72** | **0.76** | **0.60** | **0.94** |
| Bryophage | 0.00 | 0.00 | 0.00 | 0.00 | 0.00 | 0.03 | 0.00 | 0.00 | 0.00 | 0.01 | 0.00 | 0.03 |
| Florivore | 0.00 | 0.00 | 0.00 | 0.00 | 0.00 | 0.00 | 0.00 | 0.00 | 0.07 | 0.02 | 0.11 | 0.00 |
| Folivore | 0.00 | 0.00 | 0.02 | 0.00 | 0.02 | 0.00 | 0.05 | 0.00 | 0.02 | 0.01 | 0.19 | 0.01 |
| Generalist Herbivore | 0.00 | 0.00 | 0.00 | 0.00 | 0.00 | 0.02 | 0.00 | 0.03 | 0.01 | 0.07 | 0.04 | 0.01 |
| Mycophage | 0.00 | 0.00 | 0.00 | 0.00 | 0.00 | 0.00 | 0.00 | 0.00 | 0.00 | 0.01 | 0.01 | 0.00 |
| Saprophage | 0.00 | 0.00 | 0.00 | 0.00 | 0.00 | 0.00 | 0.00 | 0.00 | 0.00 | 0.01 | 0.00 | 0.00 |
| **Herbivores (All)** | **0.00** | **0.00** | **0.02** | **0.00** | **0.02** | **0.05** | **0.05** | **0.04** | **0.12** | **0.13** | **0.35** | **0.05** |
| Entomophage/Nectarivore | 0.00 | 0.00 | 0.00 | 0.00 | 0.00 | 0.00 | 0.03 | 0.00 | 0.00 | 0.00 | 0.02 | 0.00 |
| Opportunistic Granivores | 0.00 | 0.69 | 0.63 | 0.09 | 0.21 | 0.01 | 0.04 | 0.17 | 0.01 | 0.05 | 0.02 | 0.00 |
| **Omnivores (All)** | **0.00** | **0.69** | **0.63** | **0.09** | **0.21** | **0.01** | **0.07** | **0.17** | **0.02** | **0.05** | **0.04** | **0.00** |
| Detritivore | 0.00 | 0.00 | 0.00 | 0.00 | 0.00 | 0.00 | 0.00 | 0.00 | 0.00 | 0.01 | 0.00 | 0.00 |
| Carrion Feeder | 0.00 | 0.00 | 0.00 | 0.00 | 0.00 | 0.06 | 0.00 | 0.00 | 0.15 | 0.04 | 0.00 | 0.00 |
| **Saphrophages (All)** | **0.00** | **0.00** | **0.00** | **0.00** | **0.00** | **0.06** | **0.00** | **0.00** | **0.15** | **0.05** | **0.00** | **0.00** |
