# Supplementary material for: Drivers and Patterns of Ground-Dwelling Beetle Biodiversity across Northern Canada
Source: PLoS One. 2015 Apr 22;10(4):e0122163. doi: 10.1371/journal.pone.0122163 (PMC4406721; doi:10.1371/journal.pone.0122163)
Supplement: S3 Table — R-squared and significance values are determined using a permutational approach that fits each driver on the ordination space as a vector. (DOCX) [file pone.0122163.s003.docx]

| **Factors** | | **Species** | | | | **Functional** | | | |
| --- | --- | --- | --- | --- | --- | --- | --- | --- | --- |
|  |  | **Axis 1** | **Axis 2** | **R^2^** | **Pr(>r)** | **Axis 1** | **Axis 2** | **R^2^** | **Pr(>r)** |
| Climatic Factors | MeanTemp | 0.756 | 0.655 | 0.95 | **0.001** | 0.514 | -0.858 | 0.79 | ***0.024*** |
|  | MaxTemp | 0.743 | 0.670 | 0.95 | **0.001** | 0.528 | -0.850 | 0.79 | ***0.019*** |
|  | MinTemp | 0.773 | 0.635 | 0.94 | **0.001** | 0.491 | -0.871 | 0.78 | ***0.025*** |
|  | WarmMean | 0.839 | -0.544 | 0.94 | **0.002** | 0.998 | 0.060 | 0.96 | **0.001** |
|  | Coldmean | 0.497 | 0.868 | 0.92 | **0.001** | 0.310 | -0.951 | 0.70 | ***0.044*** |
|  | DDA0 | 0.995 | -0.098 | 0.96 | **0.001** | 0.867 | -0.498 | 0.94 | **0.002** |
|  | DDB0 | -0.473 | -0.881 | 0.92 | **0.002** | -0.391 | 0.921 | 0.64 | 0.076 |
|  | TotPrecip | 0.184 | 0.983 | 0.88 | **0.005** | 0.159 | -0.987 | 0.60 | 0.114 |
|  | Wind | -0.724 | 0.690 | 0.36 | 0.310 | -0.360 | -0.933 | 0.47 | 0.228 |
|  | SunHrs | 0.356 | -0.935 | 0.54 | 0.121 | 0.332 | 0.943 | 0.59 | 0.128 |
|  | Frost | 0.959 | -0.282 | 0.91 | **0.006** | 0.876 | -0.483 | 0.79 | ***0.024*** |
| Environmental Factors | SoilD | 0.173 | 0.985 | 0.20 | 0.538 | 0.065 | -0.998 | 0.74 | ***0.047*** |
|  | VarSoilD | 0.031 | 1.000 | 0.20 | 0.574 | -0.064 | -0.998 | 0.82 | ***0.015*** |
|  | MaxVegHt | 0.815 | 0.579 | 0.42 | 0.265 | 0.558 | 0.830 | 0.62 | 0.092 |
|  | GramCov | -0.509 | -0.861 | 0.67 | 0.076 | -0.309 | 0.951 | 0.80 | ***0.019*** |
|  | ShrubCov | 0.847 | 0.532 | 0.15 | 0.640 | 0.312 | -0.950 | 0.29 | 0.402 |
|  | MossCov | -0.206 | 0.979 | 0.62 | 0.094 | -0.782 | -0.623 | 0.21 | 0.590 |
|  | LichCov | 0.682 | -0.732 | 0.23 | 0.505 | 0.261 | 0.965 | 0.28 | 0.421 |
|  | ForbCov | -0.597 | -0.802 | 0.13 | 0.680 | -0.240 | 0.971 | 0.30 | 0.393 |
|  | Lat | -0.218 | -0.976 | 0.76 | ***0.048*** | -0.239 | 0.971 | 0.51 | 0.174 |

Highly significant correlations (P < 0.01) are **bold**, significant correlations (P < 0.05) are in ***bold italics.*** Abbreviations used: MeanTemp, MaxTemp, MinTemp (mean annual, maximum and minimum temperature, respectively), WarmMean, ColdMean (mean temperature of the warmest and coldest months), DDAO/DDBO (mean degree days above and below zero), TotPrecip (total annual precipitation, including snow), Wind (mean annual wind speed), SunHrs (mean annual number of hours of cloud-free sunshine), Frost (mean annual number of frost-free days), SoilD/VarSoilD (mean and variance of active soil layer depth), MaxVegHt (mean maximum height of vegetation), GramCov, ShrubCov, MossCov, LichCov, ForbCov (mean cover class of graminoids, shrubs, mosses, lichens, and forbs), Lat (latitude)
